# Whole cell biophysical modeling of codon-tRNA competition reveals novel insights related to translation dynamics

**DOI:** 10.1371/journal.pcbi.1008038

**Published:** 2020-07-10

**Authors:** Doron Levin, Tamir Tuller

**Affiliations:** 1 Biomedical Engineering Dept., Tel Aviv University, Tel Aviv, Israel; 2 The Sagol School of Neuroscience, Tel Aviv University, Tel Aviv, Israel; CPERI, GREECE

## Abstract

The importance of mRNA translation models has been demonstrated across many fields of science and biotechnology. However, a whole cell model with codon resolution and biophysical dynamics is still lacking. We describe a whole cell model of translation for *E*. *coli*. The model simulates all major translation components in the cell: ribosomes, mRNAs and tRNAs. It also includes, for the first time, fundamental aspects of translation, such as competition for ribosomes and tRNAs at a codon resolution while considering tRNAs wobble interactions and tRNA recycling. The model uses parameters that are tightly inferred from large scale measurements of translation. Furthermore, we demonstrate a robust modelling approach which relies on state-of-the-art practices of translation modelling and also provides a framework for easy generalizations. This novel approach allows simulation of thousands of mRNAs that undergo translation in the same cell with common resources such as ribosomes and tRNAs in feasible time. Based on this model, we demonstrate, for the first time, the direct importance of competition for resources on translation and its accurate modelling. An effective supply-demand ratio (ESDR) measure, which is related to translation factors such as tRNAs, has been devised and utilized to show superior predictive power in complex scenarios of heterologous gene expression. The devised model is not only more accurate than the existing models, but, more importantly, provides a framework for analyzing complex whole cell translation problems and variables that haven't been explored before, making it important in various biomedical fields.

## Author summary

mRNA translation is a fundamental process in all living organisms and the importance of its modeling has been demonstrated across many fields of science and biotechnology. Specifically, modeling a whole cell context with a high resolution has been a great challenge in the field, making many important problems un-addressable. In this study we devised a novel model, which allows, for the first time, simultaneous simulation of thousands of mRNAs, along with various bio-physical aspects that affect translation (such as codon-resolution dynamics and shared resources pool of both ribosomes and tRNAs). We demonstrated (using experimental data) that this model is more accurate than existing ones, and, more importantly, provides a framework for addressing complex translation problems (such as heterologous expression) at whole cell scale and in reasonable time. We demonstrated the model using *E*. *coli* data, but the model can be easily tailored to other organisms as well. Our model addresses an urgent unmet need for biophysically accurate whole cell translation model with resources coupling and has potential applications in many fields, including medicine and biotechnology.

## Introduction

Dozens of studies in recent years have demonstrated the advantages of using computational models of mRNA translation in basic science and biotechnology (see, for example, [1–11]). Whole cell modelling of translation is a fundamental component of synthetic and systems biology, with implications in various fields, such as biotechnology and biomedical engineering. Despite its undoubted importance, the selection of models and tools is quite limited. According to a recent review [11], several computational and mathematical models related to competition for finite resources during translation have been suggested [12–18]. However, these models commonly address only a single resource type (ribosomes) and do not include aspects such as tRNA availability. Other studies, e.g. [19], emphasize the importance of incorporating competition for tRNAs, but usually (e.g. due to computational limitations) simulate a single mRNA or a small group of mRNAs, thus inaccurately modeling a whole cell scenario, what limits their applicability. In other cases, thorough mathematical descriptions are provided, but the models are not tailored to real data and thus are not validated and are not predictive. In other words, a comprehensive model that includes all major aspects of translation (such as discrete ribosomal dynamics, various limited resources, including tRNA and ribosomes, and the affinity of tRNA-codon interactions) on a cellular level (with thousands of simultaneously translating mRNAs) currently does not exist. All the mentioned aspects are required to properly model complex scenarios related to mutations and heterologous expression, which are fundamental both for answering basic questions in cell biophysics and evolution and for various medical and biotechnological applications. In this study we aimed to bridge this gap by providing such model which is efficient enough to simulate real whole cell scenarios.

We focused on incorporation of the following two aspects in a whole cell model of translation: 1) accurate elongation dynamics at a codon resolution; 2) finite resources pools of various types. The first aspect is important because many phenomena can be analyzed and observed only when addressing the dynamics and interactions within a polysome. Furthermore, numerous biomedical implications of translation are related to mutations (such as SNPs) along the ORF (Open Reading Frame), which often cannot be addressed well with models that treat mRNA as a bulk (e.g. mean field approximations [20]). The second aspect is particularly important when considering significant changes from the native state of the cell, like in the case of heterologous expression. Such scenarios alter the resources competition profile inside the cell, leading to a change in translation resources allocation and consequently may influence the vitality of the cell [21–23]. This aspect is also important when studying the evolution of genomes as it many times induces strong evolutionary constraints.

Currently, there are no models available that fully utilize both aspects discussed above in a way that allows feasible whole cell simulations. To bridge this gap, we have developed and implemented MP-SMTM (Multiple Pool State Machine Translation Model): a novel model, based on well-established practices of translation elongation modeling and several new concepts, including the finite pools of various translation resources. We demonstrated the model on *E*. *coli* and showed that the model has not only proved to be more accurate than existing models, but, more importantly, allowed us to study codon-resolution questions on a whole cell level, what was previously impossible.

## Methods

### Whole cell translation elongation model

The proposed single-cell model operates on three levels:

#### Cell level

**Fig 1:**
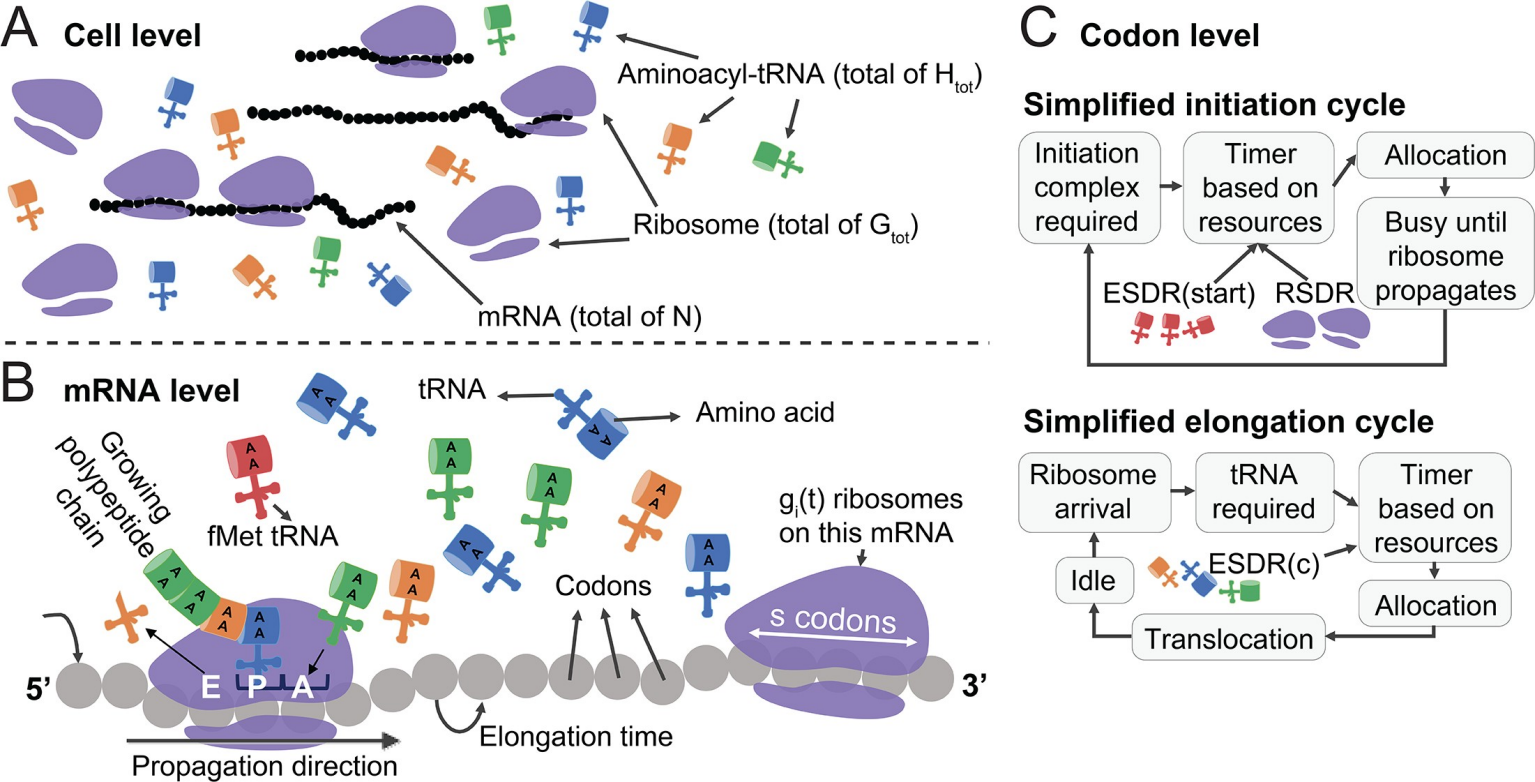
Overview of the whole cell translation model. Schematic visualization of the simulated elements at the cell level (A), at the mRNA level (B) and a simplified view of the state machines at the codon level (C), for initiation and elongation (the detailed state machines appear in F-G Figs in S1 File). At the cell level, a transcriptome of mRNA molecules with a finite pool of ribosomes and tRNAs is simulated, leading to a competition for these resources. At the mRNA level, we utilized a novel generalized deterministic TASEP model that incorporates both accurate dynamics at the codon level and dependence on cellular resources, which influences these dynamics. On the codon level, for each codon the dynamics are dictated by a state machine, an object that holds the state (e.g. “this codon is anticipating a tRNA”) and the rules of state transition order and conditions (see sub-section Generalized deterministic TASEP and state machines).

The modelled cell has a transcriptome that consists of *N* mRNA molecules, a finite ribosome pool *G*_*tot*_ and a finite tRNA pool *H*_*tot*_ (Fig 1A). Both pools are assumed to be constant in time, while their occupancy and availability change with time *t*. The amount of every mRNA corresponds to the actual mRNA level inside a real cell. There can be several independent copies of a given mRNA or a partial mRNA (S1 File), which accounts for cases with average levels smaller than one. The ribosome pool is consumed by initiation events and refilled upon termination events. At time *t* (on a timeline with resolution Δ*T*), the number of ribosomes on mRNA *i* is denoted by *g*_*i*_(*t*), while the free ribosome pool is *G*(*t*). Thus, $G_{tot}=G(t)+\sum_{i=1}^{N}g_{i}(t)=const$. The tRNA pool consists of all available tRNA types in the cell (specific to the modelled organism), with amounts corresponding to actual tRNA levels in the cell. Suppose that there are *N*_*tRNA*_ unique tRNA types. The total amount of tRNA of type *j*, *j* = 1,…,*N*_*tRNA*_, is denoted by *H*_*tot*,*j*_, while the free amount is *H*_*j*_(*t*) and the amount on an mRNA *i* is $h_{j}^{i}(t)$. Thus, the total amount of tRNA molecules is $H_{tot}=\sum_{j=1}^{N_{tRNA}}H_{tot,j}=\sum_{j=1}^{N_{tRNA}}H_{j}(t)+\sum_{j=1}^{N_{tRNA}}\sum_{i=1}^{N}h_{j}^{i}(t)=const$. We assume that upon release, tRNAs undergo aminoacylation and return to the available pool (this is also related to the aspect of tRNA recycling, which is defined in sub-section Effective supply-demand ratio).

#### mRNA level

A single mRNA molecule is modeled by a generalized version of deterministic TASEP (Totally Asymmetric Exclusion Process), described in more details in sub-section Generalized deterministic TASEP and state machines. Each mRNA is modeled as an array of codons, on which unidirectional movement (5’ to 3’) of ribosomes takes place. Ribosomes have physical size (of *s* codons) and they are not allowed to overlap or overtake one another, resulting in possible “traffic jams”. The movement of the ribosomes is modeled similarly to the current understating of the elongation cycle, including the incorporation of a suitable tRNA at the A-site, translocation and release of a non-charged tRNA from the E-site (Fig 1B). Dynamics are driven by *timers* in the following manner: when a certain step is required, the expected waiting time is calculated (according to the available resources) and a timer is assigned. In other words, a timer of a translation step is a discrete count-down of the time required for its completion. Only when the timer nullifies, the required step is performed, given all the requirements are fulfilled (otherwise, the timer is delayed). As a result, the availability of resources controls the timers, which dictate the dynamics of the system. In order to avoid resources allocation bias, at each iteration we randomized the order in which mRNAs are analyzed, resulting in uniform exposure of the mRNAs to the resources.

#### Codon level

To describe the translation elongation dynamics at a codon level, we have developed a generalized version of deterministic TASEP, which is described in sub-section Generalized deterministic TASEP and state machines.

### tRNA-codon interactions and the interaction coefficient

We used an exact, *E*. *coli* specific, codon recognition scheme [24] (Table A in S1 File), which allows wobble interactions. In order to account for the affinity of a tRNA-codon interaction (i.e. to consider the fact that a Watson-Crick interaction is more likely to happen than wobble interaction under similar conditions) we introduced an *interaction coefficient α*(*c*,*j*) between codon *c* and tRNA *j* (defined in the range (0,1]). We used similar approach to the one used in the definition of the tAI (tRNA Adaptation Index [2]), which utilizes similar coefficients to evaluate the extent to which a codon is adapted to the tRNA pool. However, we used real tRNA levels from [24] rather than tRNA gene copy number. We then defined an optimization problem and found the interaction coefficients that gave the best overall correlation between tAI and both PA (Protein Abundance) and TDR (Typical Decoding Rate, a useful translational efficiency index) [25]. Detailed steps appear in supplementary Figs A-D and Table B in S1 File.

### Effective supply-demand ratio

A fundamental aspect of translation we aimed to model is the competition for resources (such as ribosomes and tRNAs). It is known that such competition drives co-evolutionary adaptations of the organism and affects its genome (e.g. by creating codon usage bias) [1,26,27]. Furthermore, when modelling a cellular infection (e.g. with a bacteriophage) or a heterologous gene expression, the allocation of resources is expected to change, leading to a change in translational dynamics. To incorporate competition into our model, we have devised two terms (defined for each point *t* in time):

#### RSDR (Ribosomal Supply-Demand Ratio)

The ratio between the free ribosome pool *G*(*t*) (supply) and the number of mRNAs that are waiting for an initiation complex at time *t* (demand). Higher RSDR values imply lower initiation times (i.e. higher initiation rates) and vice versa.

#### ESDR (Effective Supply-Demand Ratio)

For each codon *c*, *ESDR*(*c*) represents the ratio between the number of available tRNAs that recognize *c* (supply) and the competition for these resources, namely all the codons that are recognized by these tRNAs and require a tRNA at time *t* (demand). To make ESDR as realistic as possible, we incorporated weightening based on codon-tRNA interaction coefficients and accounted for an aspect of tRNA recycling [26,28,29]; we specifically assumed that the effective demand of codon types which tend to be close to each other is lower due to recycling. ESDR was defined as:

$$ESDR(c,t)=\sum_{j\in RT(c)}\alpha (c,j)\frac{H_{j}(t)}{{\left[D_{j}(t)\right]}^{1-\exp\left(-wd_{c}\right)}},$$ (1)

where *RT*(*c*) is the group of tRNAs that recognize the codon *c* (i.e. tRNAs *j* that satisfy *α*(*c*,*j*)>0), *D*_*j*_(*t*) is the effective competition for tRNA *j*, *d*_*c*_ is the distance score of codon *c* (related to tRNA recycling) and *w* is the associated normalization factor. All arguments in the definition above are defined and described in detail in S1 File. Higher ESDR values imply lower elongation times (or higher elongation rates) and vice versa.

Both RSDR and ESDR are used continuously throughout a MP-SMTM simulation to determine the expected waiting time for a resource: when a state machine (described in sub-section Generalized deterministic TASEP and state machines) dictates that a timer needs to be defined (either for initiation or for elongation), the corresponding supply-demand ratio is calculated and the timer is defined accordingly. By doing so we assume that both supply and demand affect the waiting time. The role of the supply is more obvious. However, to justify incorporation of demand, consider, for example, a given pool of resources Y and one specific demanding object X (e.g. an initiation site X, “waiting” for ribosomes, denoted as Y). The amount of competitors (demand) can directly influence the availability of Y for X, since it affects the chance that Y will bind to a competitor, rather than to X.

In order for the model to be accurate enough to fit measured data, the estimated waiting times must be small enough, so that no significant changes will happen during this waiting time. Indeed, the error is expected to be small (up to ~3%, see S1 File). Indeed, at the regime of nominal resources, we validated that the prediction of the model in terms of protein synthesis (see sub-section ‎Prediction of protein synthesis rate and the Results section) corresponds to the available empirical protein abundance data of *E*. *coli*. It should be noted, however, that the regime of severe resources depletion is expected to be less accurate (see S1 File). The functional relations between the timers and the supply-demand ratios are given in the S1 File.

### Generalized deterministic TASEP and state machines

TASEP traditionally incorporates a one-dimensional lattice of consecutive sites and a statistical description of transition of an object (ribosome, in our case) from one site (codon, in our case) to the next one. TASEP models commonly utilize the Gillespie approach [30] for stochastic continuous-time dynamics modeling. However, since we model a large number (thousands) of TASEP-like objects coupled to a complex resources pool (that affect many of the parameters of translation), even the most efficient stochastic implementations of TASEP results in an extremely computationally intensive task. For example, stochastic TASEP simulation is estimated to take several hours and can take 6 times longer (depending in the specific parameters and CPUs) than deterministic implementation for a system with no resources coupling [11]. This ratio is expected to be much higher with pool coupling (close to two orders of magnitudes higher), since regardless of the implementation, additional factor need to be taken into account in every iteration. To avoid unreasonable running times, we took a deterministic approach. Furthermore, to incorporate accurate biophysical behavior into the model, we integrated the idea of codon state machines. Below we briefly describe the approach and provide a simple example.

In the proposed approach, each codon holds a symbolic state. The duration of each state is defined by a timer which is initiated upon state transition, according to predefined rules and up-to-date system parameters. State transition is associated with an action (e.g. ribosomal movement) and/or parameters update (e.g. ribosomal free pool increment by 1). At each simulation’s iteration, the timer is reduced by Δ*T* and only when it reaches zero, the state machine switches to the next state and the associated action takes place if possible (e.g. a ribosome is allowed to proceed only if all bio-physical conditions are fulfilled, namely the downstream ribosome is distant enough and the required resources are available). Every codon on every mRNA may have an individual timer and a state. Transition between states is monitored, and allows efficient tracking of the number of codons that require a certain resource. Detailed schemes of the state machines for both initiation and elongation are provided in Figs F-G in S1 File. A simplified version is presented in Fig 1C.

Consider, for example, the following scenario: The E site of a ribosome has translocated to a UGG codon, that now awaits a tRNA charged with Tryptophan. First, the state machine sets the state of the codon to “NeedtRNA” (the counter of “number of UGG codons that currently need tRNA” is increased by 1). Next, the state machine may require to define a timer based on the current *ESDR*(*UGG*) value. When that timer nullifies, the tRNA is allocated (if *H*_Tryptophan_>0), an amino acid bond is formed, the ribosome progresses, the tRNA is released, etc. In cases where wobble interactions may take place, the interaction coefficient *α* (sub-section tRNA-codon interactions and the interaction coefficient) is used to statistically choose the candidate tRNA.

To conclude, we kept the biophysical qualities of the TASEP model to account for realistic ribosomal movement. We used deterministic timers rather than stochastic approach for computational efficiency. The timers are defined ad-hoc according to up-to-date supply and demand metric (ESDR and RSDR). The whole process is controlled by a codon-specific state machines, which hold the state of each codon at every point in time and are the de-facto implementation of the elongation cycle.

### System parameters

The proposed model is intended for the study of various cellular conditions (e.g. viral infection) and for design and engineering modifications of existing and even new organisms. Thus, it is particularly important to simulate conditions that are as realistic as data availability permits. All prediction reported here were performed on *E*. *coli* data (K-12 MG1655 strain, downloaded from Ensembl Bacteria ASM584v2.31). Below we briefly describe how some of the parameters were obtained. Detailed description of these and other parameters, as well as their estimation methods, is provided in Figs H-I in S1 File.

#### Local initiation times

The initiation time of a given mRNA was determined by a local, mRNA-specific, value and a factor related to the global supply of initiation-related resources. Local initiation times were estimated based on ribosomal sequencing data [31]. These values where then normalized to obtain simulated ribosomal density that is in the expected range.

#### mRNAs, ribosomes and tRNAs

We maintained the relation between the number of ribosomes and number of tRNAs according to reported values [32]. We chose a representative value of 5,100 mRNA molecules, distributed according to reported mRNA levels [31]. We than performed optimization to find the size of pools that resulted in 80% ribosomal activity [32,33]. The key parameters chosen for the simulation are: *N* = 5,100; *G*_*tot*_ = 70,000; *H*_*tot*_ = 650,407; Average initiation time: 0.95*sec*. We have performed comprehensive sensitivity tests for these and other simulation parameters to make sure that the behavior of the simulation is robust and exhibits expected trends (Figs J-K in S1 File).

### Prediction of protein synthesis rate

One of the desired outcomes of a translation model is a prediction of protein synthesis rate, which is also a measure of translational efficiency. The number of termination events is a proxy to the actual number of proteins synthesized, as long as protein degradation is negligible, which is what we assume. To avoid the dependence on a specific time frame, we commonly refer to synthesis rate rather than the total number of synthesized proteins. Throughout the manuscript we use the terms termination rate and protein synthesis/production rate interchangeably. To estimate the total number of proteins synthesized, we count the total number of termination events, while taking into account the number of mRNAs of each type. We only considered termination since steady state, defined in our context as the time in which the consumption of the ribosomal pool stabilizes. PA (protein abundance) is commonly used as a proxy for the translation efficiency, and here we took the same approach, i.e. used correlations between empirical PA and simulated synthesis rate as a way to assess the predictivity of the model. We used protein abundance data from PAXdb id: 511145 (integrated dataset).

### Heterologous expression

One important property of a translation model is its usefulness in explaining a heterologous expression outcome, in part, due to its vast industrial implications. In order to asses this property, we have performed several whole cell simulations in which a heterologous gene was introduced into the *E*. *coli* cell. We used a GFP (Green Fluorescent Protein) gene from the U57608.1 cloning vector, in order to support future experimental verification. The mRNA of the GFP was inserted with a high copy-number (20% of the total mRNA molecules) and with a high initiation rate (see S1 File). The simulated cell contains both the native mRNA transcriptome and the heterologous GFPs to allow the complex interaction of pool sharing between the native and heterologous mRNAs.

Several mutated variants were simulation and compared to the original variant. Variants were based on synonymous mutations (while preserving the amino acid content) and were generated using two methods: 1) single representing codon for each amino acid; and 2) single representing codon for only one amino acid. In the first method, single codon was chosen for each amino acid based on a given (highest) score. We used the following scoring systems: TDR, mean ESDR and inverse mean occupancy. The two latter were calculated based on a simulation without a GFP gene. These scores are correlated with codon translational efficiency, thus suit as candidates for naively choosing optimal codons. The second method of variant generation is based on choosing a single codon and substituting all synonymous codons with the chosen one (leaving all other amino acids unchanged), resulting in 61 possible variants.

A landmark study of heterologous expression in the context of synonymous variants was published by Kudla *et*. *al* [34], in which they examined ~150 GFP variants (created by inducing random synonymous mutations) and their expression in *E*. *coli*. Here we aimed to show that the predictive power (in terms of protein synthesis and cell growth rate) has improved when using cell-level supply-demand considerations and specifically when using MP-SMTM. We performed a calibration procedure to properly integrate the variants into our model. Briefly, we assumed that initiation time is *τ*_*h*_ exp(−*FE*/*c*_*h*_), where *FE* is the folding energy at the beginning of the ORF, *c*_*h*_ is a calibration factor (deduced by a calibration procedure that maximizes correlation with PA, see Fig L in S1 File) and *τ*_*h*_ is some baseline value (see supplementary methods in S1 File). We then performed an independent whole cell simulation for each one of the variants, using the corresponding initiation rate and mRNA amount to study the relation between simulation predictions and empirical measurements. Finally, we used these relations to assess the prediction power of our model.

### Regressor features selection

In order to estimate the predictive power of our model, we built linear least squares multivariable regressors. In order to predict OD (Optical Density), which is related to cellular growth and PA and is an obvious prediction objective, we used features that are known to relate to translational efficiency, such as CAI (Codon Adaptation Index [35]), FE (Folding Energy) and features based on ESDR. We aimed to show that supply-demand-based features are important for accurate predictions. We took the following approach for feature selection: given a set of features *x*_1_,…,*x*_*M*_ and an objective *y*∈{*OD*,*PA*}, we performed *N*_*rep*_ = 100 repetitions of a train-test, in every one of which we iteratively chose features with decreasing importance. The training, i.e. the parameters estimation, was performed on ~67% of the variants (randomly chosen in each repetition) and tested on the other ~33%. The first feature at repetition *j*, denoted by $f_{1}^{j}$, was selected to be the one that maximized *R*^2^ (the coefficient of determination). At iteration *i*, we used features $f_{1}^{j},\ldots ,f_{i-1}^{j}$ and chose the next feature $f_{i}^{j}$ from the set $\left\{x_{1},\ldots ,x_{M}\}\backslash \{f_{1}^{j},\ldots ,f_{i-1}^{j}\right\}$ so that it maximized the *R*^2^ change. To prioritize the features for the final model, a score has been assigned as follows: if a feature *f*_*k*_ was the *i*_*j*_-th feature to be selected at repetition *j*, the score was defined as $SCR(f_{k})=\sum_{j=1}^{N_{rep}}i_{j}$. The features were then sorted ascendingly, with the lowest score being the best: $\left({\bar{f}}_{1},\ldots ,{\bar{f}}_{M})=sort(SCR(f_{1}),\ldots ,SCR(f_{M})\right)$.

### Additional terminology

The term occupancy of a codon stands for the number of simulation iterations for which a ribosome was located on the codon. Similarly, occupancy profile simply refers to the location dependent occupancies of a series of codons, such as an ORF. *Ribo-seq* stands for ribosome sequencing or ribosome profiling, which is an experimental method of estimation of ribosomes location at the codon level [36]. In processed ribo-seq results, each codon on each mRNA hold a read count value, which is related to the chance that a ribosome is located in this codon and to the mRNA level (among other factors). *CUB* stands for Codon Usage Bias, namely the extent to which the distribution of synonymous codons differs from an expected uniform distribution. When a quantitative representation of CUB was needed, we used CAI [35] (see supplementary methods in S1 File for the calculated values for *E*. *coli* codons).

## Results

We have performed several analyses with the aim of showing the usefulness and accuracy of MP-SMTM. In short, we showed high correlations between simulated and empirical values (sub-section High correlation between model predictions and experimental measurements), demonstrated the effect of codon order on translation efficiency (sub-section ‎Estimation of the effect of codon composition and order on translation efficiency) and provided a thorough case study of heterologous gene expression, while showing the importance of competition for resources (sub-section Competition for tRNAs explains variations in heterologous translation). In all cases we used empirical *E*. *coli* data.

### High correlation between model predictions and experimental measurements

**Fig 2:**
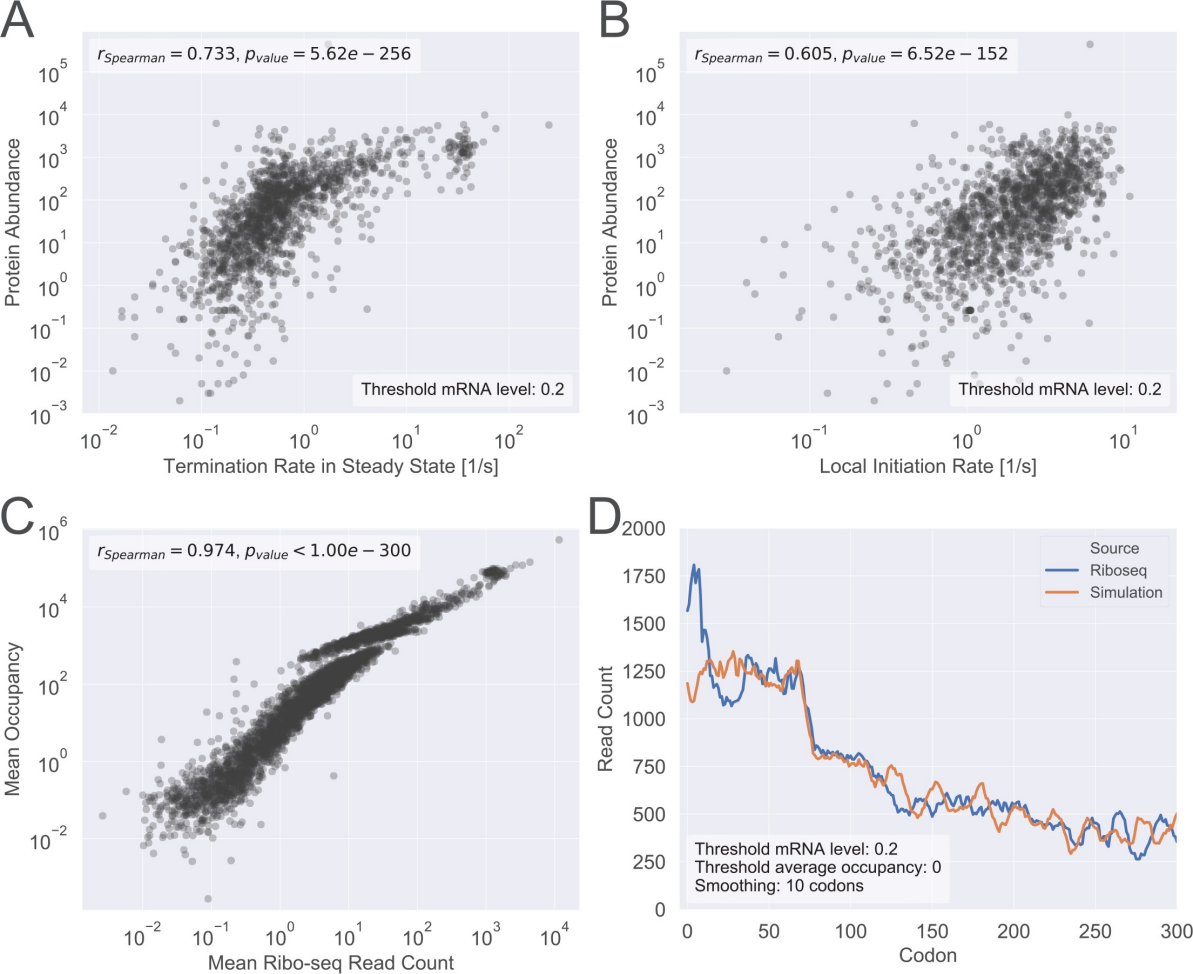
High correlation between the predictions of the MP-SMTM model and measured data for *E*. *coli*. (A) Termination rate in steady state (predicted by the model) and protein abundance (empirical data); (B) Local initiation rate (as estimated for the model) and protein abundance (empirical data); (C) Mean ribo-seq read count and mean simulated occupancy. (D) Ribosomal density profiles for both simulation (average occupancy per codon) and ribo-seq (average read count per codon). In (A), (B) and (D) mRNAs with level lower than 0.2 were omitted from this analysis to avoid discretization errors of the simulation. In (A), (B) and (C) each point represents a single mRNA type. (All terms in this figure are defined in the Methods section, sub-sections System parameters, Prediction of protein synthesis rate and Additional terminology).

As a first validation step, we correlated the predictions of our model with various experimental gene expression measurements. We observed a high Spearman correlation of *r* = 0.733, *p*_*value*_ = 5.62×10^−256^ between the simulated termination rate and the empirical protein abundance (Fig 2A). This value is higher than previous state-of-the-art PA predictors [2,20,37]. Due to the fact that translation tends to be an initiation rate limited process (leading to a high correlation between initiation rate and termination rate), one may argue that the correlation observed in Fig 2A is explained solely by initiation rate. This is, however, not the case, since the correlation between the local initiation rate and the protein abundance is only *r* = 0.605, *p*_*value*_ = 6.52×10^−152^ (Fig 2B). Thus, the initiation rate explains ~37% of the protein level variance, while the complete model (initiation + elongation) explains ~54% of the variance. We conclude that incorporating aspects of elongation into the model indeed improves its predictions.

We then compared the mean occupancies to the mean ribo-seq read count (obtained from [31]) and obtained an exceptionally high correlation of *r* = 0.974, *p*_*value*_<10^−300^ (Fig 2C), even though the correlation between the read count and the corresponding estimated local initiation rate is *r* = 0.554, *p*_*value*_ = 1.81×10^−281^ (Fig M in S1 File). Lastly, we qualitatively compared the simulated average ribosomal profile with ribo-seq results. Specifically, we calculated the average occupancy (for the simulation) and the average read count (for the ribosomal sequencing), per codon, across the entire transcriptome. In both cases (Fig 2D) it is evident that the first 50–100 codons exhibit higher ribosomal density, which is with agreement with previous reports [27].

### Estimation of the effect of codon composition and order on translation efficiency

It was suggested that codon order and composition are strongly related to the translation efficiency [26,38,39] as it expected to affect ribosomal dynamics and thus protein synthesis. Translation efficiency can be assessed using the total rate of protein synthesis, which is expected to be related to growth rate. However, the previous studies in the field failed to show that indeed there is a direct effect of codon usage and order on growth rate and did not provide any evaluation of the magnitude of this effect.

**Fig 3:**
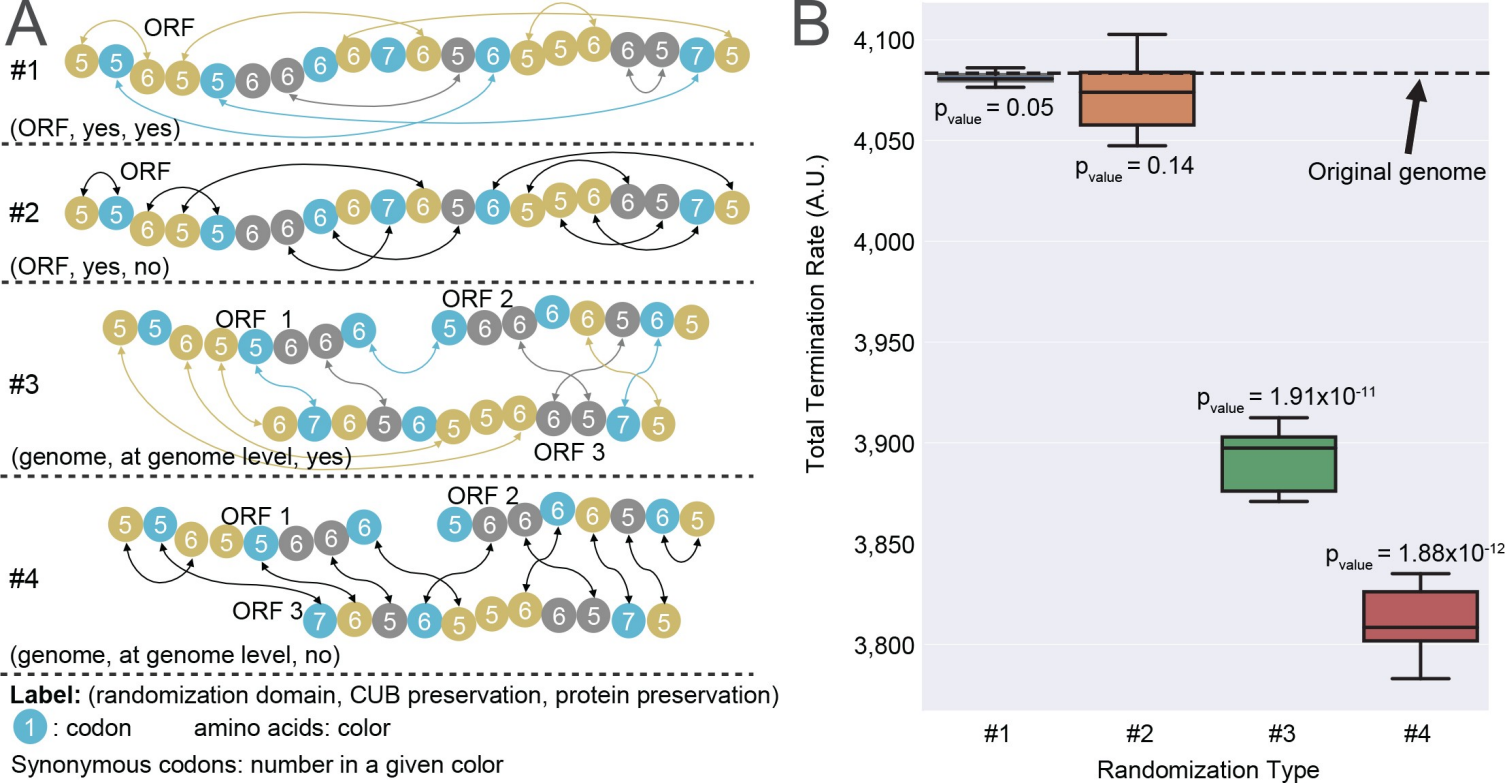
The effect of the codon order and composition on the total termination rate. (A) Schematic illustration of the different randomization types. (B) Box plot for total simulated termination rate (i.e. the sum of termination rates of all mRNAs) distributions (10 values each) compared to the un-randomized scenario. The p-values shown are the result of a single-sample two-sided t-test.

To provide initial answers to these questions we have performed 4 types of random modifications to the genome (Fig 3A), with different combinations of CUB and amino acid preservations within each ORF or across the entire genome. Each type was randomized 10 independent times. The total protein synthesis rate was calculated for each case and compared to the un-randomized baseline using a single-sample two-sided t-test. We observed a significant change in translation rate both for the local and the global randomizations (Fig 3B). Naturally, in the cases where the CUB was maintained at the ORF level, a small decrease in the total termination rate was observed. In the cases where CUB was only maintained at the genome level, the total termination rate has been reduced significantly more.

### Competition for tRNAs explains variations in heterologous translation

**Fig 4:**
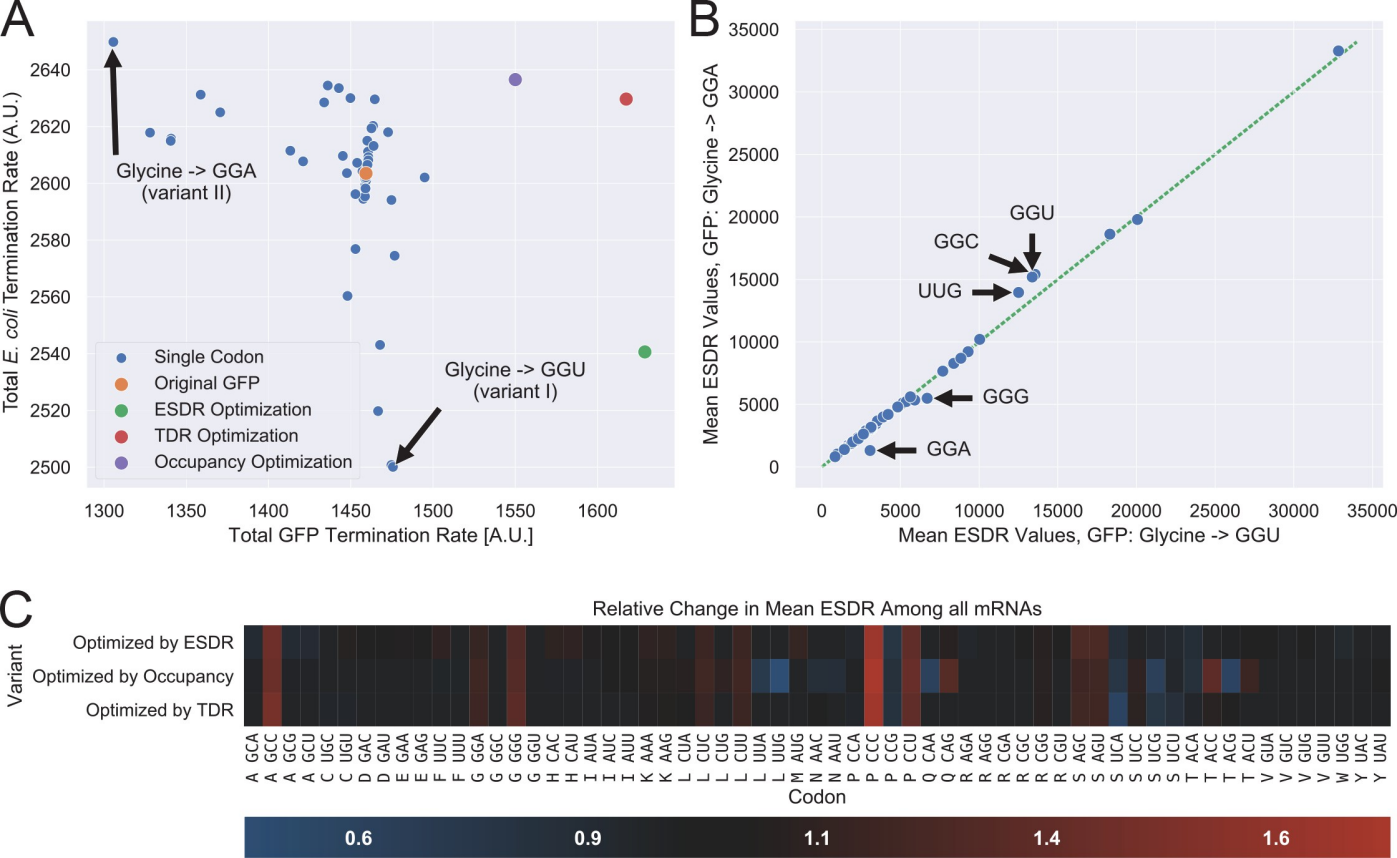
The model promotes understanding and provides analysis framework for heterologous expression problems. (A) Total *E*. *coli* and GFP genes termination rates for various GFP variants as heterologous genes. Two method were used for variant generation, as described in the sub section Heterologous expression in the Methods. The original variant is shown in orange. Smaller blue dots are related to variants in which one codon was chosen as substituted all synonymous codons (61 such variants in total). Other dots represent variants in which a single representing codon was chosen for each amino acid, according to some optimality score (either TDR, ESDR or inverse occupancy). (B) Two variants (marked with arrows in (A)) are compared in terms of ESDR. Codons for which the ESDR is different represent changes in supply and demand of associated tRNAs and allow to understand the results in (A). (C) Comparison of three optimization variants in terms of their ESDR, per codon, relative to the unoptimized variant (red represents a higher ESDR value). Some codons (such as CCC) exhibit an increase in ESDR in all variants, indicating that increasing the supply/demand for these codons can improve overall translational efficiency.

We have examined the total protein synthesis rates (of *E*. *coli* and GFP) for the two variant generation methods (as described in the methods sub-section Heterologous expression) (Fig 4A). It can be seen that the values for 61 variants of method 2 are distributed around the values of the original variant, showing the optimization potential. Not surprisingly, the more rational variants of method 1 (which is based on scoring codons per amino acid) led to a higher GFP synthesis rate. Let us discuss two specific variants and demonstrate how ESDR provides a useful analysis framework. Two variants (generated using method 2) are marked with an arrow: a variant in which Glycine is represented solely by GGU (variant I) and a variant in which the same amino acid is represented by GGA (variant II). Interestingly, these two variants exhibit very distinct results. We can observe the mean ESDR values of all codons for these two variants and compare them, as shown in Fig 4B. Most of the ESDR values are similar in both variants, but those that don’t demonstrate a possible reason for the different behavior. Relative to variant I, the amount of free tRNA-UCC in variant II is lower, resulting in lower ESDR of GGA. Since tRNA-UCC is also recognized by GGG, its ESDR also drops in variant II relative to variant I. On the other side, codon-GGU is only recognized by tRNA-GCC. This tRNA is expected to be more abundant in variant II (since it is no longer demanded by GGU codons in the GFP mRNAs), what indeed increases the ESDR of codon GGU and codon-GGC, the other codon that this tRNA recognizes. Furthermore, we can see an increase in the ESDR of UUG, which cannot be easily explained with the reasoning just provided. Such analysis shows what resources are important for the native *E*. *coli* genes and for the GFP gene. For example, the availability of tRNA-GCC is important for the *E*. *coli* genes, since its reduction resulted in decreased protein production.

We have compared the three variants generated by method 1 in Fig 4C, which depicts relative changes in ESDR for all codons, grouped by amino acid. Interestingly, all variants indicate that the ESDR of CCC, GCC, CCU (and to a lower extent GGG, AGC and AGU) should be increased in order to increase the overall GFP synthesis. On the other side, the ESDR of UCA and UCG should be decreased.

We then performed model validation using results published by Kudla et. al [40]. In order to show that the model provides better predictivity of both PA and OD relative to other common approaches, we have calculated several correlations and built various relevant regressors. We have considered FE, *N*_*mRNA*_ (the number of mRNAs, as reported by Kudla *et*. *al*), CAI and two results of the simulation: TR (translation rate of the GFP mRNAs) and %AR (percent of active, i.e. translating, ribosomes). We have considered only variants with complete data availability. The TR was found to be highly correlated to the PA (*r*_*Spear*_ = 0.66, *p*_*value*_ = 4.28×10^−11^ and *r*_*Pears*_ = 0.66, *p*_*value*_ = 2.77×10^−11^, Fig N in S1 File). The FE, which was claimed to be highly correlated with PA as well, exhibited a slightly lower correlation of *r*_*Spear*_ = 0.63, *p*_*value*_ = 4.14×10^−10^ and *r*_*Pears*_ = 0.63, *p*_*value*_ = 5.83×10^−10^.

When linear regression was used for PA prediction, the FE underperformed the proposed model. Indeed, linear regressor based on FE gave *r*_*Spear*_ = 0.62, *p*_*value*_ = 2.36×10^−9^ and *r*_*Pears*_ = 0.62, *p*_*value*_ = 1.6×10^−9^ while TR gave *r*_*Spear*_ = 0.64, *p*_*value*_ = 2.44×10^−10^ and *r*_*Pears*_ = 0.65, *p*_*value*_ = 1.76×10^−10^ (in all cases, the correlation is between the prediction objective and the prediction). A multivariable linear regressor with both FE and TR gave *r*_*Spear*_ = 0.73, *p*_*value*_ = 3.14×10^−14^ and *r*_*Pears*_ = 0.73, *p*_*value*_ = 5.71×10^−14^, i.e. 37% improvement in the explained variance relative to FE regressor. CAI failed to predict PA (*r*_*Spear*_ = 0.13, *r*_*Pears*_ = 0.08). Details for all the regressors are provided in the S1 File.

In terms of OD prediction, FE, as expected, did not perform well (*r*_*Spear*_ = −0.01,*r*_*Pears*_ = 0.06). CAI predicted OD better than TR (*r*_*Spear*_ = 0.6, *p*_*value*_ = 8.15×10^−9^ and *r*_*Pears*_ = 0.53, *p*_*value*_ = 6.96×10^−7^ for CAI). However, the model provided additional valuable information, since the best multivariable regressor turned to be based on CAI, TR and %AR, with *r*_*Spear*_ = 0.64, *p*_*value*_ = 3.28×10^−10^ and *r*_*Pears*_ = 0.73, *p*_*value*_ = 7.55×10^−14^. The relation between %AR and OD is not surprising, since higher % of free ribosomes means more polysomes in the cell cytoplasm available for translating the endogenous genes, leading to increased growth rate.

We hypothesized that the higher accuracy of MP-SMTM is related to the supply and demand of resources, or more specifically, to the ESDR. The multitude of variants was sufficient to observe how ESDR correlates positively with PA (or OD) for some codons, but negatively for others. To isolate the codon-specific effect, we simply calculated the correlation between the PA (or OD) and the mean ESDR values across all variants, for each codon. To ensure that the effect is not the result of CUB or the FE, we controlled for these two variables. In the PA case, 8 codons exhibited negative correlation of −0.40<*r*_*Spear*_<−0.19 and 4 codons exhibited positive correlation of 0.1<*r*_*Spear*_<0.3 (while $p_{value}<\frac{0.05}{61}$, i.e. 5% with a strict Bonferonni correction). The OD case showed similar numbers (all results are available in S1 File). The asymmetry between positive and negative correlations is not surprising, since increase in ESDR of a codon (resulted in an increased average decoding rate) is more likely to cause a ribosomal traffic jam and decrease translational efficiency than the opposite scenario.

**Fig 5:**
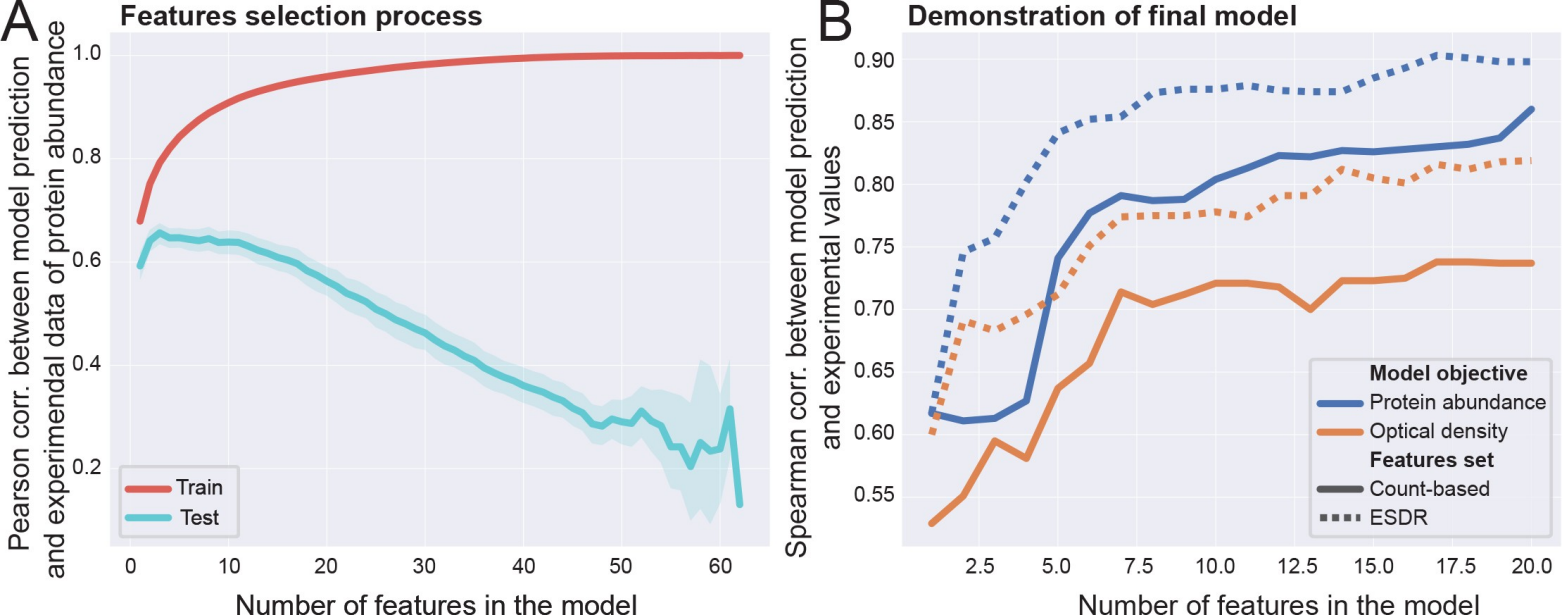
A multivariable regressor for predicting protein abundance (PA) and optical density (OD) of GFP variants. (A) Pearson correlation between model prediction and the measured PA values of the GFP variants, as function of the number of feature selected for the train and the test sets. This graph demonstrates the approach taken for feature selection: For 100 times, a train (~67%) and test (~33%) sets were randomly selected. Each time, the next best feature was selected to be the one the increases *R*^2^ the most in the test set (for more details, see sub-section 'Regressor features selection' in the Methods section). This result suggests that a model with more than ~10 features will show poor predictivity due to over-fitting. (B) After choosing the best features and sorting them, this figure shows the correlation between the predicted PA (blue) and OD (red) values and the measured ones, for increasing number of features. A features' set based on ESDR (continues line) was compared to a simpler metric of codon-count (dashed lines). In both cases (PA and OD) the ESDR-based model performed better and reached impressive correlation with empirical data, demonstrating the importance of our model (Data for this model was taken from Kudla *et*. *al* [34]).

The fact that ESDR can explain PA and OD at the codon level suggests that some codons can serve as regressor features for PA and OD predictions. We defined 64 features (ESDR of 61 codons and the start codon, FE and CAI) and performed feature selection using the approach described in the Methods sub-section Regressor features selection, resulting in a prioritized list of features $\left({\bar{f}}_{1},\ldots ,{\bar{f}}_{64}\right)$. The result of such process is demonstrated in Fig 5A. For evaluation, we have compared the results to an alternative model, resulted from an alternative set of features that can be calculated per codon per variant–namely the number of occurrences of a codon at a given variant. The comparison was done using the entire data set, with the features $\left({\bar{f}}_{1},\ldots ,{\bar{f}}_{k}\right)$ for increasing number of *k* for each model. As seen in Fig 5B, for both PA and OD, the ESDR-based model has outperformed the alternative model, achieving an impressive correlation (the lists of chosen features for all regressors are given in Figs O-R in S1 File).

## Discussion

We have presented, for the first time, a framework that allows not only accurate translation elongation modeling at the codon level, but also incorporation of various whole cell aspects such as competition for resources (both ribosomes and tRNA). The main challenge was to build a model that not only captures all the mentioned aspects, but is also efficient enough to allow real whole cell simulations with real parameters, such as thousands of mRNAs undergoing translation at the same time (a task which various other studies failed to accomplish). Moreover, the formulation of this framework as a system of generalized deterministic TASEP objects allows relatively easy generalization: it is possible to incorporate additional factors to further refine the model when additional experimental data will be available.

The chosen model organism, *E*. *coli*, proved to be useful to establish the fundamentals of the model and simulation as there are many available relevant gene expression measurements for this organism that can be used for inferring the model parameters. However, similar framework can be utilized to simulate Eukaryotic cells and even multi-cellular organisms. Such modification will require formulation of different state machines (that may have additional signals as an inputs) and a relevant codon-tRNA recognition table (to account for the organism-specific interactions that can also include non-standard amino acids), but the basic principle of a system of generalized TASEP objects remains.

We have demonstrated that the model can be used for solving fundamental problems such as predicting the outcome of heterologous gene expression, both in terms of protein synthesis rate and cellular growth. We have shown that observing ESDR at the codon level is extremely beneficial in such prediction problems. Specifically, we have shown that a multivariable regressors based on codon-specific ESDR features can reach Spearman correlation of as high as 0.8 with protein abundance (and 0.7 with optical density), outperforming models with traditional features such as folding energy and codon adaptation index. We have also demonstrated various case studies in which the model can be harnessed to gain a deeper understanding of the complex supply and demand dynamics in the cell.

The importance of studying supply and demand of tRNA has already been demonstrated before [1,10,19]. The relation between tRNA abundance and codon decoding rates was previously modeled [41,42] and various aspects of tRNA aminoacylation and recycling dynamics [19,43–45]. Despite all these advances, our model is currently the first that considers the biophysics of tRNA supply and demand at a cellular level with thousands of mRNA molecules performing translation simultaneously, while accounting for wobble interactions, finite pool of tRNAs and ribosomes, ribosomal dynamics, and tRNA recycling while maintaining correspondence to real empirical data.

The devised model has several additional advantages. The codon resolution allows using this model for complex medical and biomedical engineering problems. For example, various types of cancers are associated with mutations that affect protein synthesis rate. This model allows examining the effect of possible therapeutic mechanisms and their effect on the behavior of the cell. Furthermore, the model allows planning and predicting the outcome of complex experimental setups, allowing significant time optimization when such assays are performed.

Nevertheless, as in any model, several limitations should be discussed. First, all simulations presented in this paper assumed constant resources pools. However, it is known that these may vary due to various cellular conditions, division, external stimuli or stress. For simulating non-constant resources all relevant parameters (e.g. the values of tRNA, mRNAs, and ribosomes over time, DNA replication rates, etc) are needed. When the right parameters are can be estimated, modeling a time-variable pool is possible within the suggested framework of the current model, and should be thoroughly considered; this can be done for example via assumption and simulation of pseudo steady state of the system. Similarly, translation is known to be coupled to additional aspects such as DNA replications and cell growth that can be added to our model when high resolution measurements of these processes are available.

Additionally, the model is not expected to behave accurately is scenarios with severe resources depletion. Since the discussed organism was *E*. *coli*, it worth mentioning that co-transcriptional translation is known to occur [46], but we did not incorporate this aspect into the model. Additional aspects that were not taken into account (mainly due to high complexity and/or lack of reliable experimental data) are mRNA/tRNA degradation and cell-division (which is, by itself, a very challenging process to model, involving DNA replication and various steps of the cell cycle). With that said, a previous study showed that for a simple degradation model, the protein levels should be correlated with predicted translation rate even when omitting degradation [20]. Finally, the model is in-part deterministic and does not include all the stochastic aspects of translation. These aspects should be addressed in a future study.

To conclude, we believe that MP-SMTM is a meaningful step towards accurate whole cell modeling of translation. This work opens the door to many more advances in the field, and allows further progress in the utilization of such modelling and analyzing important problems in medicine, synthetic biology, biotechnology and biomedical engineering.

## Supporting information

S1 File The main supplementary file, including supplementary methods, results and details. (PDF)

S2 File Correlation between PA (or OD) and ESDR per codon. (XLSX)
