# Supplementary material for: Whole cell biophysical modeling of codon-tRNA competition reveals novel insights related to translation dynamics
Source: PLoS Comput Biol. 2020 Jul 10;16(7):e1008038. doi: 10.1371/journal.pcbi.1008038 (PMC7375613; doi:10.1371/journal.pcbi.1008038)
Supplement: S1 File — (PDF) [file pcbi.1008038.s001.pdf]

SUPPLEMENTARY MATERIALS

1. Partial mRNAs

For mRNAs with abundance greater than 0.5, the value is rounded to the closest integer and the system is initiated with an integer number of these mRNAs, each acting as an individual mRNA object. For relatively low levels, this discretization error may be significant at the individual mRNA level, e.g. if the real abundance is 1.5, but it is rounded down to 1, there is a 50% difference in the representation of this mRNA. For the whole system simulation this error is negligible, but when further analysis is performed, we can insert an ad-hoc correction, assuming that total number of terminations of an mRNA type is proportional to its abundance.

When mRNAs with abundance lower than 0.5 are considered, a different approach is taken. Every such mRNA is represented by a single mRNA object labeled by ‘Partial mRNA’. Such objects act as regular mRNA object with one difference: upon initiation, there is an additional randomization that allows initiation only in the fraction of times equal to the abundance. Such implementation results in partial consumption of resources (and consequently partial number of synthesized proteins), representing a fractional abundance. Clearly, this is an approximation because such effective reduction in initiation rate results in different ribosomal densities. However, because such mRNAs are rare by definition, and rare mRNA tend to have lower initiation rates, we expect that the approximation is reasonable.

2. E. coli tRNA-codon recognition scheme

**Anti-Codon**	**AA Name**		**Codons**	**Comments**	**Molecules count from** (1)
UGC	Ala1B	A	GCU,**GCA**,GCG	GCU (W-), CGC (W+)	3250.00
GGC	Ala2	A	**GCC**	GCU (#), but is recognized by UGC tRNA	617.00
AGC	Ala	A	(*)		
CGC	Ala	A	(*)		
ACG	Arg2	R	**CGU**,CGC,CGA	CGC and CGA (W+)	4752.00
CCG	Arg3	R	**CGG**		639.00
UCU	Arg4	R	**AGA**	AGG (#), but recognized by CCU tRNA	867.00
CCU	Arg5	R	**AGG**		420.00
GCG	Arg	R	(*)		
UCG	Arg	R	(*)		
GUU	Asn	N	**AAC**,AAU	AUU (W+)	1193.00
AUU	Asn	N	(*)		
GUC	Asp1	D	**GAC**,GAU	GAU (W+)	2396.00
ATC	Asp	D	(*)		
GCA	Cys	C	**UGC**,UGU	UGU (W+)	1587.00

ACA	Cys	C	(*)		
UUG	Gln1	Q	**CAA**	CAG (#) but recognized by CUG tRNA	764.00
CUG	Gln2	Q	**CAG**		881.00
UUC	Glu2	E	**GAA**, GAG	GAG (W+)	4717.00
CUC	Glu	E	(*)		
CCC	Gly1	G	**GGG**	comment 1	1068.00
UCC	Gly2	G	**GGA**, GGG	GGG (W+)	1068.00
GCC	Gly3	G	**GGC**, GGU	GGU (W+)	4359.00
ACC	Gly	G	(*)		
GUG	His	H	**CAC**, CAU	CAU (W+)	639.00
AUG	His	H	(*)		
GAU	Ile1	I	**AUC**, AUU	comment 2, AUU (W+)	2084.00
LAU	Ile2	I	AUA	comment 3	1390.00
AAU	Ile	I	(*)		
UAU	Ille	I	(*)		
CAG	Leu1	L	**CUG**		4470.00
GAG	Leu2	L	**CUC**, CUU	CUU (W+)	943.00
UAG	Leu3	L	**CUA**, CUG	CUG (W+)	666.00
CAA	Leu4	L	UUG		1913.00
UAA	Leu5	L	**UUA**, UUG	UUG (W+)	1031.00
AAG	Leu	L	(*)		
UUU	Lys	K	**AAA**, AAG	AAG (W+)	1924.00
CUU	Lys	K	(*)		
CAU(fMet)	Met f1 + f2		**AUG** (start codon)	comment 4	1926.00
CAU	Met m		**AUG**		706.00
GAA	Phe	F	**UUC**, UUU	UUU (W+)	1037.00
AAA	Phe	F	(*)		
CGG	Pro1	P	**CCG**		900.00
GGG	Pro2	P	**CCC**, CCU	CUU (W+)	720.00
UGG	Pro3	P	**CCA**, CCU, CCG	CCG (W+), CCU (W-)	581.00
AGG	Pro	P	(*)		
UCA	Sec	U	**UGA**	comment 5	219.00
UGA	Ser1	S	**UCA**, UCU, UCG	UCG (W+), UCU (W-)	1296.00
CGA	Ser2	S	**UCG**		344.00
GCU	Ser3	S	**AGC**, AGU	AGU (W+)	1408.00
GGA	Ser5	S	**UCC**, UCU	UCU (W+)	764.00
ACU	Ser	S	(*)		
AGA	Ser	S	(*)		
GGU	Thr1 + Thr3	T	**ACC**, ACU	comment 6, ACU (W+)	1199.00
CGU	Thr2	T	**ACG**		541.00
UGU	Thr4	T	**ACA**, ACU, ACG	ACG (W+), ACU (W-)	916.00
AGU	Thr	T	(*)		
CCA	Trp	W	**UGG**		943.00

GUA	Tyr1 + Tyr2	Y	**UAC**,UAU	comment 7, UAU (W+)	2030.00
AGA	Tyr	Y	(*)		
UAC	Val1	V	**GUA**,GUG,GUU	comment 8, GUG (W+), GUU (W-)	3840.00
GAC	Val2A + Val2B	V	**GUC**,GUU	comment 9, GUU (W+)	1265.00
AAC	Val	V	(*)		
CAC	Val	V	(*)		

Table A: Breakdown of recognition scheme below. All codons associated with a given anti-codon are taken from (1), with the Watson-Crick pairs emphasized with bold.

(W-) is not a classic wobble interaction
(W+) is a classic wobble interaction
(#) should be allowed according to classic wobble interaction, but not allowed in this case
(*) Not listed in (1), in agreement with zero gene copy number in http://gtrnadb.ucsc.edu/genomes/bacteria/Esch_coli_K_12_MG1655/

Comment 1: this isoacceptor was counted with Gly2 (indistinguishable spot in the 2D gel). Their total tRNA count (2,136) was divided equally between CCC and UCC with respect to a gene copy number of 1 and 1, respectively.

Comment 2: this isoacceptor was counted with Ile2 (indistinguishable spot in the 2D gel). Their total tRNA count (3,474) was divided in ration of 3/2 between GAU and CAU with respect to a gene copy number of 3 and 2, respectively.

Comment 3: AUA is recognized by the anticodon 5'-LAU'-3', when L is Lysidine, a nucleoside that undergoes transformation from cytidine (carbonyl is replaced by the amino acid lysine). In (1) this anti codon is marked as "CAU".

Comment 4: The tRNA count indicated as Met f1 and Met f2 correspond to the same tRNA and codon, so their count (1211 and 715, respectively) was combined. Also, even though the canonical start codon is AUG, other start codons exist (E. coli K-12 uses 83% AUG, 14% GUG, 3% UUG and few others (2). All these codons, if located at the start location, are recognized by fMet-tRNA.

Comment 5: Selenocysteine (also known as Sel-Cys) is the 21th proteinogenic amino acid. Unlike other amino acids, selenocysteine is not coded for directly in the genetic code. Instead, it is encoded in a special way by a UGA codon, which is normally a stop codon. Such a mechanism is called translational recoding. Again unlike the other amino acids, no free pool of selenocysteine exists in the cell. Instead, cells store selenium in the less reactive selenide form (H2Se). Selenocysteine synthesis occurs on a specialized tRNA, which also functions to incorporate it into nascent polypeptides. The primary and secondary structure of selenocysteine-specific tRNA, tRNASec, differ from those of standard tRNAs in several respects. The selenocysteine tRNAs are initially charged with serine by seryl-tRNA ligase, but the resulting Ser-$tRNA^{Sec}$ is not used for translation because it is not recognized by the normal translation elongation factor (EF-Tu in bacteria).

Comment 6: The tRNA count indicated as Thr1 and Thr3 correspond to the same tRNAs and codons, so their count (104 and 1095, respectively) was combined.

Comment 7: The tRNA count indicated as Tyr1 and Tyr2 correspond to the same tRNAs and codons, so their count (769 and 1261, respectively) was combined.

Comment 8: In (1) the anti-codon was indicated as “UAG”, although this anti-codon recognized Leucine. This is probably a mistake, so it was converted to UAC.

Comment 9: The tRNA count indicated as Val2A and Val2B correspond to the same tRNAs and codons, so their count (630 and 635, respectively) was combined.

The Pearson and Spearman correlations between the estimated number of tRNA molecules and the corresponding gene copy number are 0.73 and 0.64 with $p_{value} < 1e-5$ in both cases.

3. CAI (Codon Adaptation Index) table

We used the library CodonAdaptationIndex (https://github.com/Benjamin-Lee/CodonAdaptationIndex), along with the supplied list of highly extressed genes in *E. coli*. We then calculated the CAI values for each codon (3), resulting in the following values:

Codon	CAI	Codon	CAI	Codon	CAI	Codon	CAI
AGG	0.0027	AGT	0.1235	GCC	0.5079	CAC	1.0000
ATA	0.0042	TGA	0.1357	CGC	0.5277	CAG	1.0000
CGG	0.0053	TCA	0.1441	TAT	0.5313	CCG	1.0000
AGA	0.0063	CCA	0.1791	TGT	0.5502	CGT	1.0000
CTA	0.0078	AAT	0.1884	GTG	0.7104	CTG	1.0000
CGA	0.0087	ACG	0.1986	GCA	0.7247	GAA	1.0000
TAG	0.0090	CAA	0.2101	AGC	0.8191	GAC	1.0000
CCC	0.0158	TCG	0.2191	GCT	0.8266	GCG	1.0000
GGA	0.0332	AAG	0.2432	GAT	0.8479	GGT	1.0000
TTA	0.0354	GAG	0.2882	TCC	0.8662	GTT	1.0000
TTG	0.0527	GTC	0.3257	GGC	0.8695	TAA	1.0000
CTT	0.0592	CAT	0.3970	AAA	1.0000	TAC	1.0000
ACA	0.0686	TTT	0.4024	AAC	1.0000	TCT	1.0000
GGG	0.0719	ATT	0.4865	ACC	1.0000	TGC	1.0000
CTC	0.0905	ACT	0.4918	ATC	1.0000	TGG	1.0000
CCT	0.1213	GTA	0.4919	ATG	1.0000	TTC	1.0000

4. Interaction coefficients optimization problem

When considering codon-tRNA interactions, we should consider both the canonical Watson-Crick (WC) pairs and other possible wobbling interactions. Section 2 lists all possible interactions in *E. coli* K-12 strains, along with the measured tRNA abundances. We define ***Interaction Coefficient*** (IC) as the chance, relative to WC pairs, that a binding between a codon (that currently resides at the A site of a ribosome) and an aminoacyl tRNA will take place, given that the tRNA is located in the vicinity of the codon. By this definition, the IC of WC crick pairs is 1, while other wobbling pairs will have an IC of less than 1 (but not less than zero).

Below we estimate the IC of all non-WC pairs by means of the tRNA Adaptation Index (tAI) (4). Similarly to the definition of Reis et al., we define the tAI of a codon c:

$$tAI(c) = tAI(codon) = \sum_{i \in RT(c)} a_{X:Y} N_i$$

Where N_i is the number of tRNA molecules available in the cell, while $a_{X:Y}$ is the IC between an anti-codon whose first (from 5') nucleotide is X and a codon whose third nucleotide is Y. This index is a measure of the extent to which a codon is adapted to the tRNA pool; e.g. a codon that is recognized by a relatively abundant tRNA pool will have a relatively higher score.

For a given set of IC values, a tAI for all codons can be calculated. Then, these values are normalized by the maximum tAI value. The tAI of a gene is defined as the geometrical mean of all normalized tAI scores of the ORF codons (including the start codon but excluding the stop codon).

In order the estimate the IC, we adapt the following strategy: we first find the set of IC that will maximize the correlation between the tAI and the TDR scores of the codons (5). Assuming there are several candidates (with similar correlation), we choose those that will maximize the correlation between tAI gene scores and their protein abundances of all *E. coli* genes. The idea is to avoid overfitting. The procedure is schematically described below:

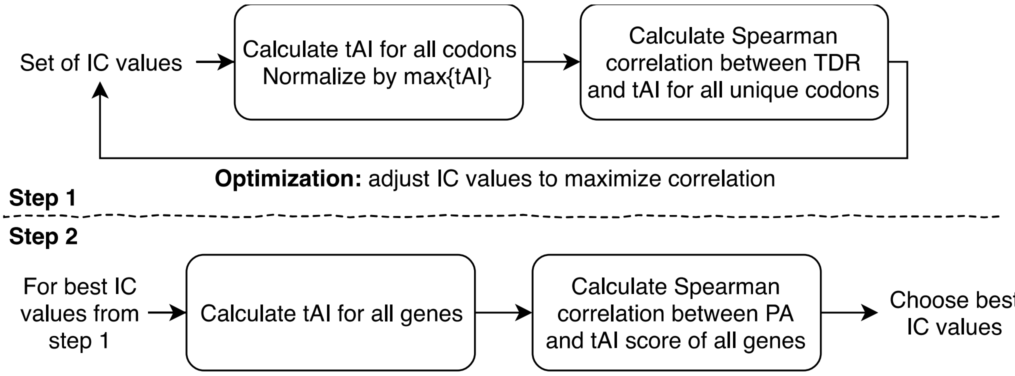

Fig A: Schematic description of the optimization process of the interaction coefficients (IC). In step 1, a given set of IC is optimized to reach maximal correlation between TDR and tAI. In step 2, the best IC values are used to calculate the correlation between PA and tAI. The best IC set is chosen.

We estimate the IC vector, which contains the following six parameters:

$$\vec{a} = (a_{A:A}, a_{A:C}, a_{G:U}, a_{U:G}, a_{U:U}, a_{L:A})$$

Use the following pipeline for optimization:

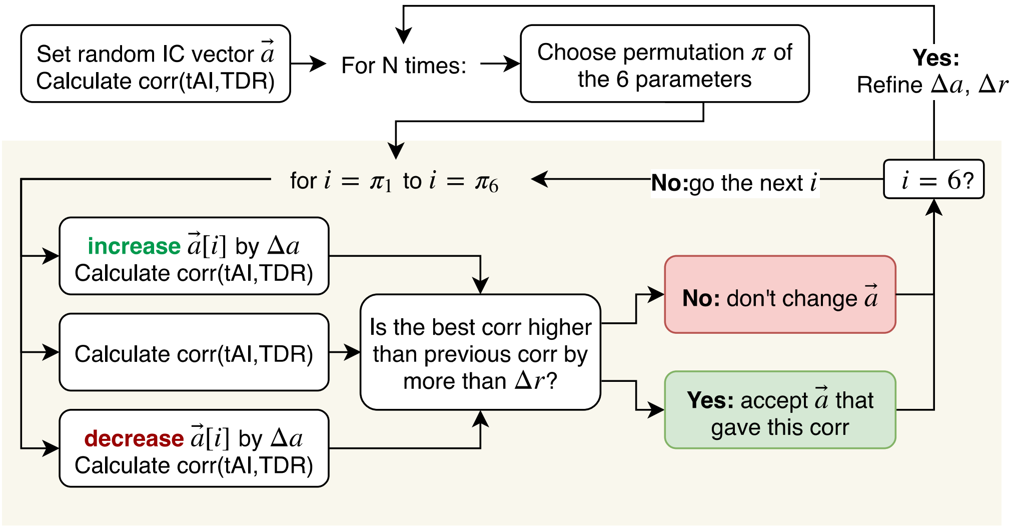

Fig B: Schematic description of the "hill climbing" procedure in step 1 of the optimization.

The whole procedure was perform 50 times for different initial vectors $\vec{a}$. We used $N = 20$ and perform 4 iterations, each time with $\Delta a = 0.1$ that is decrease 10-fold each iteration (up to $\Delta a = 0.0001$), while $\Delta r = 0.001$ for all iterations. Below a violin plot of all final $\vec{a}$ vectors:

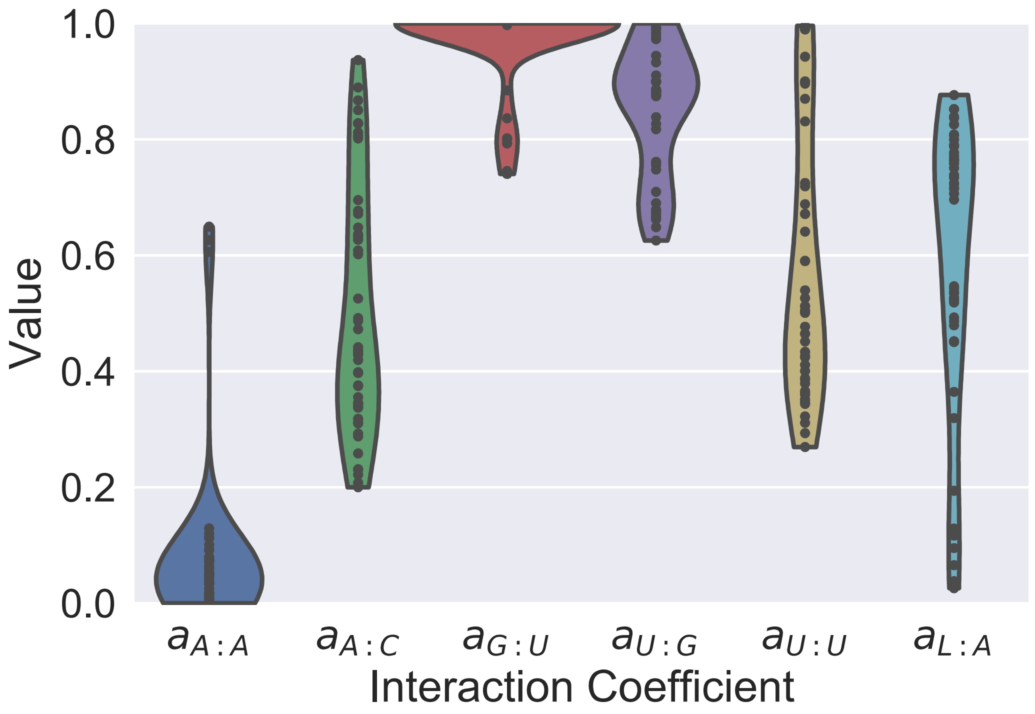

Fig C: Violin plot of the best IC sets from step 1. It is evident that some coefficients, such as $a_{G:U}$ are more constrained than others.

The final correlation in all cases is between 0.484 and 0.549 with $p_{value} < 1e - 5$ in all cases. There are some $\vec{a}$ candidates with similar high correlation. In order to avoid overfitting, we use all $\vec{a}$ candidates to calculate the tAI gene scores and get the correlation with PA (all $p_{value} < 1e - 240$).

Below, we can see a scatter of these correlations. We chose the vector that gives best agreement with both TDR and PA, as shown in the figure.

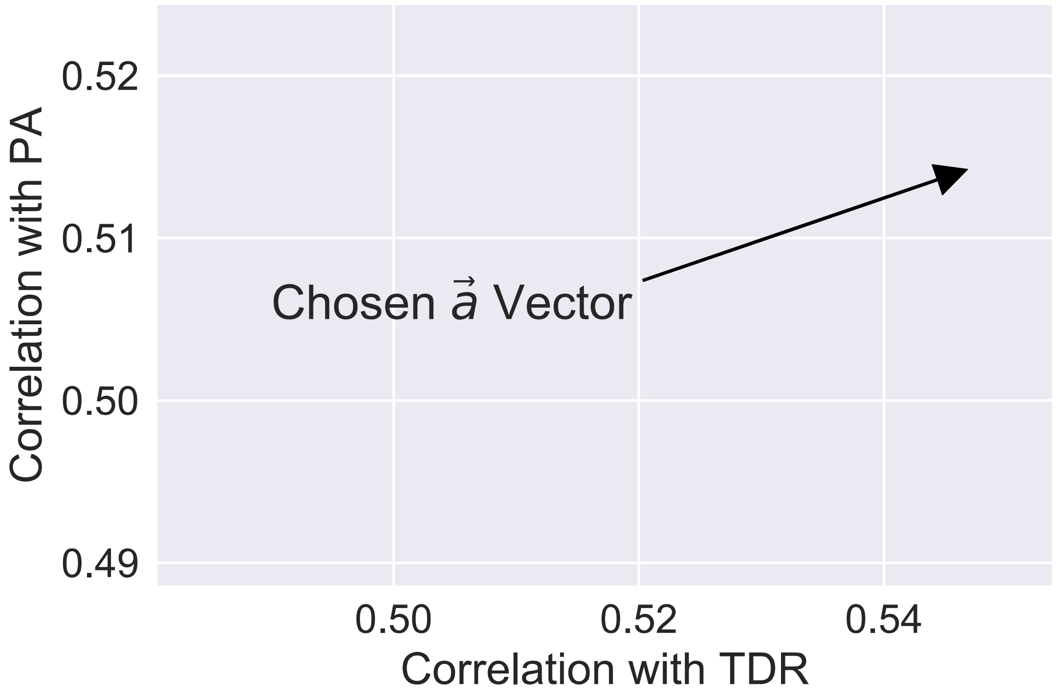

Fig D: The final selection of the IC set. Every IC set is represented by a point, where the size of a point corresponds the p_{value} of the correlation with TDR (larger = lower p_{value}). The chosen set it shown with an arrow.

We note that during optimization $a_{A:A}$ has reached a values of 0.0000, making this interaction impossible. Increasing this values up to 0.02 does not decrease the discussed correlation in the chosen resolution. We also note that the 4th decimal digit does not affect the correlation.

The chosen vector is:

$$\vec{a} = (a_{A:A}, a_{A:C}, a_{G:U}, a_{U:G}, a_{U:U}, a_{L:A})$$

$$= (0.020, 0.200, 1.000, 0.662, 0.366, 0.547)$$

This vector gives Spearman correlation of 0.547 with TDR ($p_{value} = 5e - 6$) for all codons, and 0.514 with PA ($p_{value} = 3e - 272$). For all *E. coli* genes.

Below the tAI values calculated for all codons, sorted from the lowest to the highest value:

CGA	0.0194	CCC	0.1466	CGC	0.1935	ACC	0.2442	UGC	0.3232	GAC	0.4879	GAA	0.9605
UGA	0.0446	CAA	0.1556	UUA	0.2099	UCG	0.2448	UGU	0.3232	GAU	0.4879	CGU	0.9676
AGG	0.0855	UCC	0.1556	UUC	0.2112	UCU	0.2522	GGG	0.3614	GUG	0.5176	CUG	1.0000
CCA	0.1183	AGA	0.1765	UUU	0.2112	GUC	0.2576	AAA	0.3918	UUG	0.5285		
GCC	0.1256	CAG	0.1794	AUA	0.2162	AAG	0.2594	start	0.3922	GUU	0.5438		

CAC	0.1301	ACA	0.1865	GGA	0.2175	CCG	0.2616	UAC	0.4134	GAG	0.6359	
CAU	0.1301	CCU	0.1899	ACG	0.2336	UCA	0.2639	UAU	0.4134	GCA	0.6618	
CGG	0.1301	CUC	0.1920	GCU	0.2422	AGC	0.2867	AUC	0.4244	GUA	0.7819	
CUA	0.1356	CUU	0.1920	AAC	0.2429	AGU	0.2867	AUU	0.4244	GGC	0.8876	
AUG	0.1438	UGG	0.1920	AAU	0.2429	ACU	0.3124	GCG	0.4381	GGU	0.8876	

Table B: tAI values of the final IC set.

5. Detailed definition of ESDR and functional relations with timers

tRNA-codon recognition scheme that allows various interaction for each codon, with different interaction coefficients and tRNA abundances, induces complexity on the resource management of the model.

Ribosome pool: Consider first the case of the ribosome pool. As there is only one type of ribosome, the rate of initiation events is related to the availability of free ribosomes (supply) and the current competition for these ribosomes (demand). Thus, upon initiation we can simply calculate the *Ribosomal Supply-Demand Ratio* (RSDR), and use this value to calculate the initiation timer.

tRNA pool: The case for tRNAs, however, is more complex. For a given codon, we assume that the penalty waiting time is tightly related to the effective competition on the resources. For a given codon c at a given time, we check what are the available tRNA molecules that recognize this codon, and weight them by interaction coefficient α. However, the demand for a resource must be also taken into account. We define the *Effective Supply-Demand Ratio* for codon c as follows:

$$ESDR(c) = \sum_{j \in RT(c)} \alpha(c,j) \frac{H_j}{D_j^{1-\exp(-wd_c)}}$$

where H_j is the currently available pool of tRNAs of type j, $RT(c)$ is the group of tRNAs that recognize the codon c (i.e. tRNAs j that satisfy $\alpha(c,j) > 0$) and

$$D_j = \sum_{k \in RC(j)} \tilde{\alpha}(k,j) N_{req,cod}(k)$$

is the *demand*, the effective number of codons can be recognized by the tRNA of type j. Note that both H_j and D_j are time dependent. Here $RC(j)$ is the group of codons that are recognized by tRNA j (i.e. codons c that satisfy $\alpha(c,j) > 0$), $N_{req,cod}(k)$ is the total number of codons of type k that currently require a tRNA and $\tilde{\alpha}$ is normalized α, as defined below. The power of D_j, namely $1 - \exp(-wd_c)$, represents a penalty related to tRNA recycling, with d_c being a *distance score* of codon c (defined below) and w is a normalization factor to be estimated below. The rational is as follows: if two codons that are recognized by a given tRNA are adjacent to one another along the ORF, there is increased probability that the same tRNA will also deliver an amino-acid to the downstream codon, leading to effectively lower demand. It should be mentioned that the tRNA that exists the E ribosomal site is not charged with an amino acid, and it takes some time for the tRNA to undergo aminoacylation.

However, we still expect some allocation bias which is related to the distance between codons that share the same tRNA type. The parameter w, which may be biophysically related to the aminoacylation time, is in fact a way to control the magnitude of the effect (extremely short aminoacylation times relative to decoding times will result is higher recycling rate, while extremely long aminoacylation times will result in no recycling at all). As will be described below, we estimate its value based on biophysical considerations.

The suggested penalty related to the average distance between codons. For each codon c, we calculate the average distance, in codons, between instances of c, across all ORFs in the transcriptome, normalized by the mRNA level. Formally, let ORF i have a corresponding mRNA abundance of L_i. Let $\left\{l_1^c, l_2^c, \dots l_{k_i}^c\right\}$ be an ordered group of locations of codon c in this ORF. If $k_i \leq 1$, the ORF is not considered. The distance score d_c is defined as:

$$d_c = \frac{\sum \frac{L_i}{k_i} \sum_{j=2}^{k} l_j^c - l_{j-1}^c}{\sum L_i} = \frac{\sum \frac{L_i}{k_i} \left(l_j^c - l_1^c\right)}{\sum L_i}$$

In order to estimate w we take the following approach: TDR, much like ESDR, reflects the effective competition among codons and tRNAs. Thus, both metrics are a measure of codon optimality (or adaptation to the tRNA pool) and should be correlated. We prefer using TDR over tAI because the former is estimated using experimental data and more reliably captures the dynamic information of translation elongation. ESDR itself is a dynamic metric, and its calculation requires a running whole-cell simulation, making optimization very time consuming. As an alternative, for this optimization we use naïve ESDR. It is defined similarly to the ESDR, but instead of considering only currently available tRNAs and competing codons, we consider all relevant tRNAs and codons.

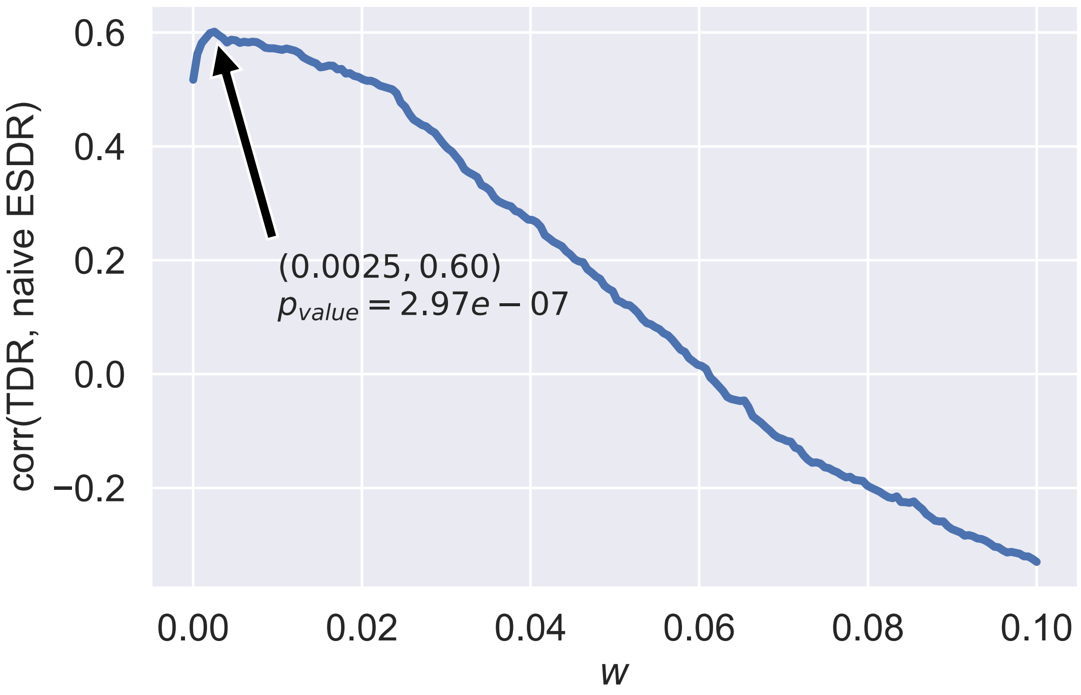

Fig E: Optimization of the normalization coefficient w. The correlation between TDR and naïve ESDR was calculated for various values of w. Interestingly, the value needs to be small enough in order for the recycling mechanism to take place. Specifically, the value chosen is the one that maximizes the correlation.

Note that for high w values the correlation is negative. This is related to the fact that more optimal codons are also more frequent, leading to a higher demand. The denominator of the ESDR is correlated stronger to TDR than the nominator, leading to inverse correlation. Accounting for effective tRNA recycling as described above helps to overcome this anomaly.

Normalization of α: the normalized interaction coefficient is defined by

$$\tilde{\alpha}(k,j) = \frac{\alpha(k,j)}{\max\{\alpha(c,j) | c \in RC(j)\}}$$

It is the interaction coefficient between codon k and tRNA z, normalized by the maximum affinity between the tRNA j and any codon it recognizes. The normalization exists to avoid cases in which a given tRNA j satisfies $\alpha(c,j) < 1 \; \forall \, c \in RC(j)$, leading to increased supply/demand ratio for this particular tRNA.

Choosing the tRNA: assuming that the timer for waiting for tRNA at codon c has passed, we try to allocate a tRNA out of the currently free ones. The probability of choosing $j \in RT(c)$ will be proportional to the affinity times the amount of the given tRNA, namely $\alpha(c,j)H_j$.

6. mRNA-Pool Interaction

The interaction between mRNAs and the cell resources is modelled through the timers that dictate ribosomal movement. The timer expresses the estimated time for the required resource to arrive, depending on pool availability. Timers are defined in the following cases:

Initiation: the canonical prokaryotic initiation mechanism is relatively well understood. Local, mRNA-specific, factors include the distance of the Shine-Dalgarno sequence from the start codon, fold energy at this region, energy released upon initiator tRNA hybridization etc. These factors essentially determine *local initiation time*. We address the estimation of local initiation times in section 9. In addition, global factors such as the availability of ribosome, initiator tRNAs and various initiation factors may significantly alter the actual initiation time. While modeling only ribosome and tRNA pools, we define initiation timers using the following expression:

$$\tau_{init} = \tau_0 \left[1 + (\gamma - 1) \exp\left(-\frac{ESDR(start)}{2\beta}\right) \exp\left(-\frac{RSDR}{2\delta}\right)\right]$$

Here τ_0 is the local initiation time. $ESDR(start)$ is the ESDR of start codons (associated solely to fMet). The maximal possible timer is $\gamma\tau_0$, for cases where both supply/demand ratios approach zero. Finally, β and δ are simply normalization factors that control the steepness of the relation. The value of γ significantly influences time penalty only when the system operates near depletion of resources, which is not the case in real cells. We assign a default value of 10. The value of β is addressed below. The default value of δ is 1. We perform sensitivity tests to both γ and δ.

When such timer reaches zero and assuming a ribosome was allocated successfully, the first codon after the start codon will be located at the A site of the ribosome, starting the first elongation cycle.

Elongation: Similarly to the initiation step, we refer to the canonical model of the translation elongation cycle. Our model assumes that the duration of each cycle can be viewed as sum of two components:

(1) A constant asymptotic time, which includes all steps under the assumption of infinite resources. This component is codon-independent.
(2) A variable time, which reflects the limited availability of the tRNA pool (and thus codon-dependent). We assume that the variable part only depends on the ESDR of the codon being translated. In other words:

$$\tau_{elongation} = \tau_{asym} + \tau_{\mathrm{MPRET}} \exp\left(-\frac{ESDR}{\beta}\right)$$

Here τ_{asym} is the asymptotic constant time, which is the only term in the case resources saturation ($ESDR \to \infty$). The term $\tau_{\mathrm{MPRET}} \exp\left(-\frac{ESDR}{\beta}\right)$ is the penalty due to the limited resource pool (MPRET stand for maximal pool-related elongation time), which can be at most τ_{pen} when $ESDR = 0$. The parameter β reflects the 'dynamic range' of the ESDR dependence, i.e. what ESDR change is required to make a given change in the total time.

A partial justification for such pool-based timers is a well-established claim is that codons that are better adapted to the tRNA pool have better translational efficiency (6).

We now define typical values for τ_{asym}, τ_{pen} and β. Suppose the average codon decoding times for all codons are given: $T_1, T_2, \ldots, T_{61}$. We assume that the lowest values correspond to most abundant tRNAs and thus $\tau_{asym} \approx \min\{T_i\}$. On the other side, we assume that the slowest codons correspond to low $ESDR$, leading to $\tau_{MPRET} \approx \max\{T_i\} - \min\{T_i\}$ (approximately 100ms). Finally, in order to evaluate β, we make the following claim: elongation time depends on the ESDR in such a way, that differences in ESDR values are maximally expressed as differences in the resulted time. This simply reflects the assumption that the functional expression of elongation time must be overall maximally sensitive to adaptation to the tRNA pool (which we model via ESDR). One approach is to define that the median ESDR value results in an elongation time halfway between τ_{asym} and $\tau_{asym} + \tau_{MPRET}$. Thus,

$$\tau_{asym} + \tau_{\text{MPRET}} \exp\left(-\frac{median(ESDR)}{\beta}\right) = \tau_{asym} + \frac{\tau_{MPRET}}{2} \rightarrow \beta = \frac{median(ESDR)}{ln2}$$

Note that we treat β as a normalization factor for ESDR for all codons. In order for this normalization to be stable, the value must reach steady state (otherwise, the normalization factor may fluctuate an induce non-physical artifacts to the model). We show that β indeed reaches steady state.

To estimate the constant part, we follow (7) that considers variation of translocation time for various concentrations of EF-G and Mg2+. For in vivo compatible concentrations ($\sim 10\mu M$ EF-G and $\sim 1mM$ Mg2+), the translocation time was roughly 31ms. This value of 30ms can be taken as the constant translocation time τ_{asym}.

Termination: We take a simple approach to termination, modeling it as a single step process of constant time. The time is estimated using ribosomal sequencing and described below.

Timer error estimation: typically, a timer will be assigned and only when it finished – the associated action will take place if possible. For example, consider assignment of timer for tRNA allocation. As defined above, the penalty that is related to resources is smaller or equal to $\boldsymbol{\tau_{\text{MPRET}} = 100ms}$. Assuming $\boldsymbol{\Delta = 5ms}$, we expect not more than 20 iterations until the timer ends. In this time, based on a simulation of native *E. coli* with nominal parameters (as estimated below), we expect $\boldsymbol{ESDR}$ change of not more than 20% and $\boldsymbol{\beta}$ change of not more than 5%. With typical values being $\boldsymbol{\beta \approx 4,000}$ and lowest $\boldsymbol{ESDR}$ values of around 700 (for CCA), we expect an error of the timer value of around 3%. Note that this represents a very extreme case.

The regime of severe resources depletion: When ESDR approaches zero, we assign timer value of $\tau_{asym} + \tau_{\text{MPRET}}$ (that were tailored based on experimental data) at most. By using this approach, we avoid cases in which a temporary low ESDR results in an extremely high timer (which is problematic if the depletion is resolved while the timer is still with high value). The potential problem with such approach is that for very low ESDR values, the timer is biased to the selection of τ_{asym} and τ_{MPRET}

values, regardless of the discussed codon. In all the cases we tested, such limitation did not raise any problems (in part because severe resources depletion is not a realistic condition, while we used only empirical data). It should be emphasized, that any regime in which resources are not depleted – the suggested approach is a good approximation, as demonstrated by the high correlations with empirical data.

7. Detailed state machines

The state machines below are presented graphically and implemented in the mRNA.py file.

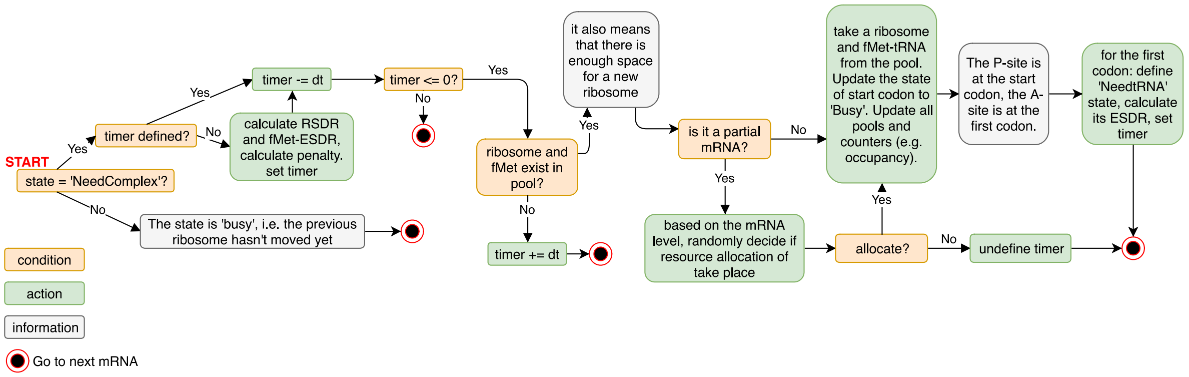

Fig F: Schematic representation of the initiation state machine. Such logic is utilized by every start codon in the system.

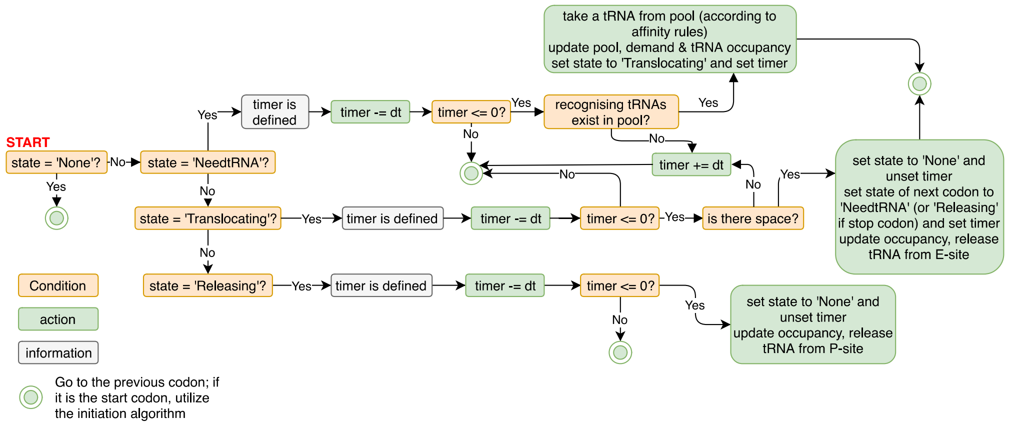

Fig G: Schematic representation of the elongation state machine. Such logic is utilized by every codon in the system, except from the start codon – for each another state machine is defined.

It should be noted that addressing codons in reverse order (starting from 3' to 5') is done for computational efficiency (allowing to avoid several conditions), but the expected error for such implementation (which appears also in previous studies) is very low. The induced error from such biased order of timer allocation is very small. As explained above (section 6, "Timer error estimation"), we can expect an error of less than 3% in the allocated timer over 17 iterations of 5ms, thus less than 0.2% in a single ΔT. This error represents, in part, inaccuracies in the demand that result from biased

order of timer definitions. It may be argued that addressing codons in such order may result in incorrect allocation of resources. However, in additional to the small error, it should be further noted that such scenario is rare in the first place, since it required several codons on the same mRNA that require the same tRNA to start in finish decoding within ΔT=5ms. With tens of ribosomes translating a typical mRNA (~20 codons per ribosome, and hundreds of codons forming a typical ORF) that consists of tens of codon types, such scenario is indeed rare at the whole-cell landscape.

8. Model parameters and estimation methods

Nominal Parameters - Method

In this section we describe the way we chose nominal parameters for the mode, i.e. the set of parameters use by default for all reported results (unless otherwise mentioned).

We should distinguish between biological parameters and mathematical parameters. The first kind includes, for example, the number of mRNA molecules, number of ribosomes and tRNA etc. These values must be consistent with known reported values of the studied organism, which is *E. coli* in our case. The mathematical parameters, such as the β and δ in the SDR functions, are proprietary for the presented model and must only satisfy consistent predictions and robustness. In a section 11, we show that the model is robust for parameters change to the expected extent.

We took the following approach (schematically depicted below):

1. We chose a value of 5,100 for the number of mRNAs in the cell (this is a typical representative value, see the next sub-section).
2. We performed a whole cell simulation for a range of ribosome pool values (denoted here as R) and a range of average local initiation times values (denote here as I). For a given set of (R, I):
    a. We calculated the number of tRNA molecules that corresponds to R (assuming linear relationship and values from (8)).
    b. We obtained a set of local initiation times, estimated individually for each mRNA (details in a section 9). These values were normalized to have an average of I (so that the estimation yielded the relative values).
3. For each such simulation, the following values were calculated:
    a. The percentage of active ribosomes at steady state. This values is expected to be 80% (8).
    b. The average number of codons per active ribosome (equivalent to distance between ribosome centers). This values is expected to be 26 – 14 codons/ribosome for growth rates of 0.6 – 2.0 doublings/hour (8), respectively.

All values for these simulations are discussed in the section “Nominal Parameters - Values”.

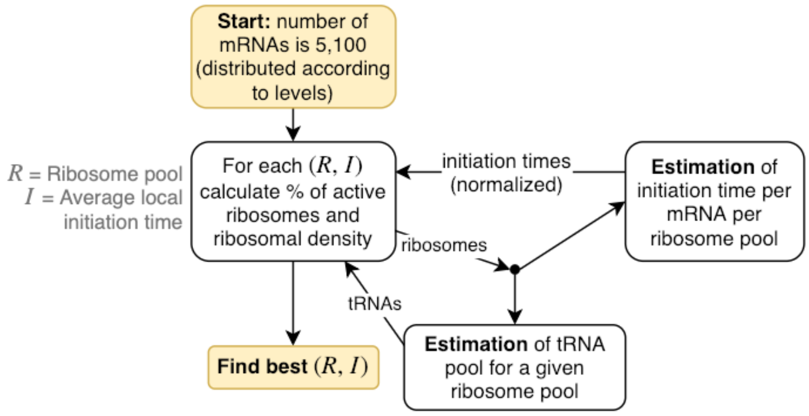

Fig H: Schematic description of the process of estimating the main system parameters, namely the size of the ribosome pool, average local initiation time and the size of tRNA pool.

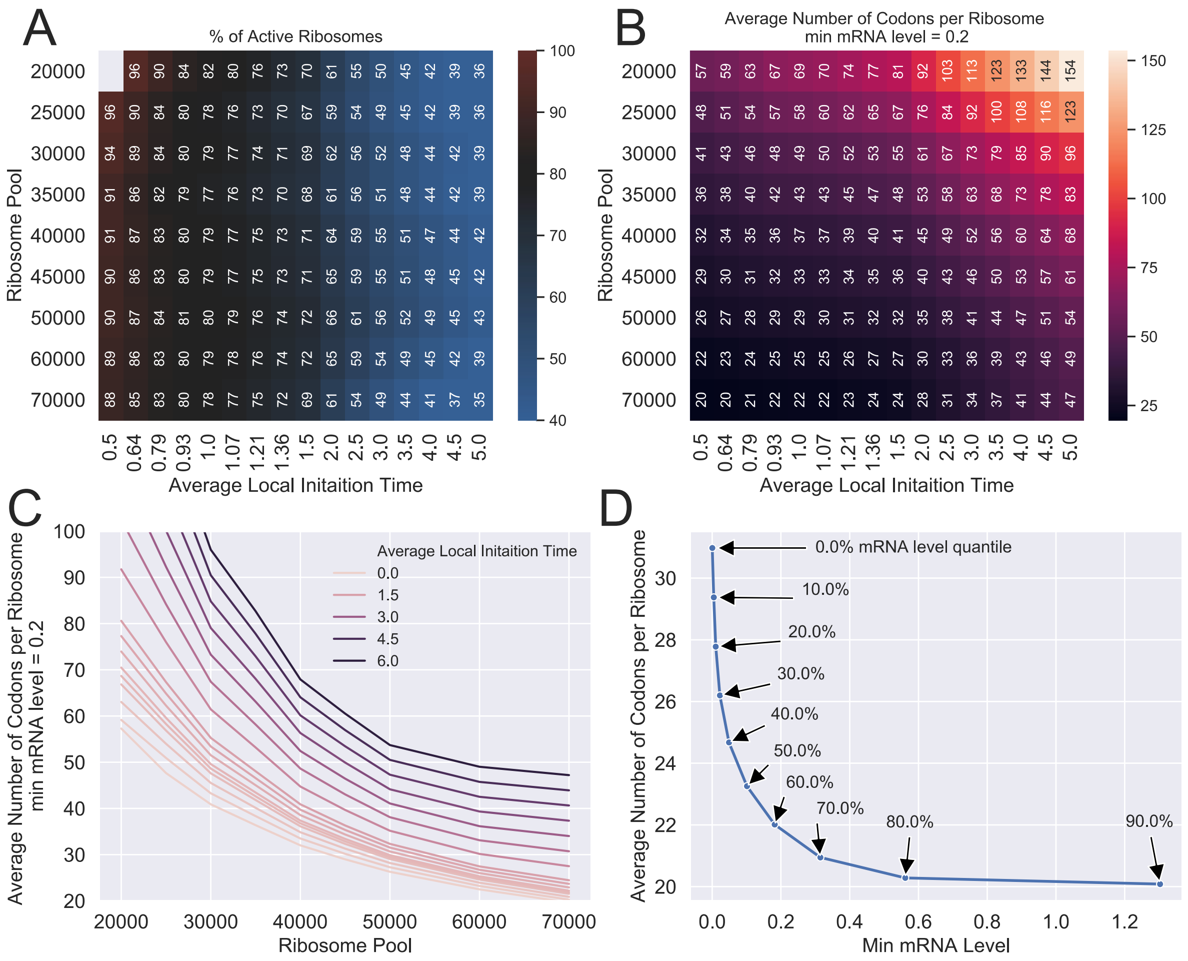

Fig I: Estimation of nominal system variables. (A) Matrix of ribosome pool values and average initiation time values. Only some values agree with the required ~80% ribosomal activity, which is represented by color. (B) Same as A, but the shown value is the average number of codons per ribosomes (or the average distance between ribosomes). (C) Same data as B, with different representation. The values are shown for mRNAs with level higher than 0.2. (D) Justification to the

selected threshold of 0.2: at this value, the average number of codons per ribosome begins to saturate.

It should be noted that ideally, for a given growth rate (resulted from a well-defined *E. coli* strain and growth medium), it should be possible conclude the number of ribosomes, tRNA and mRNA molecules. However, The growth rate is not the only explaining factor and moreover, all provided measurements are not synchronized in conditions. For this reason we adopted a more robust optimization method as described above.

<u>Nominal Parameters - Values</u>

Transcriptome:

1. Coding sequences: Coding sequences for *E. coli* K-12 MG1655 strain were downloaded from: Ensembl Bacteria (ASM584v2.31). From a total number of 4,497 transcripts, only 4,140 are coding genes (those that contain an ORF). Each of these genes was treated as an individual mRNA even though in some genes are originated in operons and thus result in polycistronic mRNAs. The rest, that includes small non-coding genes (e.g. miRNA, rRNA etc.) and pseudogenes (had evolutionary history with functioning genes but have been mutated and are no longer functional), have been omitted. Out of the 4,140 coding genes, 49 were excluded due to various reasons, while the remaining 4,091 are used in the model.
2. Relative mRNA levels: (differential expression) Both relative expression levels and deep ribosomal profiling were performed by Li et. al (9).
3. Absolute number of mRNA molecules: From (10) we concluded a value of 7,800 for rapid growth in LB medium, and 2,400 for moderate growth rate in MM. However, explicit growth rate is not reported. From (11) follows that *E. coli* in similar mediums reaches a growth rate of 0.7 – 2 doublings/h.
   More crude analysis (BNID111919 (12)) shows that there should be 1,000-10,000 mRNAs in bacterial cell.
   These values were also validated in (13) when modelling the rate of RNA chain elongation.

tRNA Pool:

1. Relative number of tRNA molecules: we used (1) to identify the relative amounts of different tRNA molecules in *E. coli.* See Table A for the values used.
2. Absolute number of tRNA molecules and ribosomes: (8) reports that the estimated number of tRNA molecules is 63,000 - 669,000 for growth rates of 0.6 – 2.0 doublings/hour. Values that are deduced from (1) indicate a similar range (when taking into account the provided ribosomes/tRNAs ratio and range of ribosome pool values from (8)).

Ribosomes Pool: reports that the estimated number of tRNA molecules is 6,800 - 72,000 for growth rates of 0.6 – 2.0 doublings/hour. These numbers show consistency for various estimation methods (BNID111527 (12)).

Initiation Time: Translation initiation time is considered to be on the order of seconds in bacteria (14). This order of magnitude is with agreement with some specific reported values, such as 3 sec of the wild-type lacZ mRNA of *E. coli* (15). Also, assuming the previously mentioned distance of 14 – 26 codons between ribosome, and assuming that elongation time is 5 – 22 codons/sec (see “estimation of local initiation times”), we conclude that, on average, a new ribosome begins translating a given mRNA roughly every 1 - 5 seconds. Unfortunately, more precise values at the mRNA-specific resolution are not available. We used the range of 0.5 – 5.0 sec for parameters estimation.

9. Estimation of local initiation times

Below the method of initiation time estimation the for all *E. coli* genes.

Suppose we have N types of mRNA molecules, where type $i \in \{1, \ldots, N\}$ has mRNA level of M_i.

For each mRNA type i we denote read count by $RC_i = \left(RC_0^i, RC_1^i, \ldots, RC_{L_i-1}^i\right)$, where RC_j^i is the read count at codon j and L_i is the number of codons in the ORF, including the start and stop codons. The total read count for this mRNA type:

$$TRC_i = \sum_{j=0}^{L_i-1} RC_j^i$$

The total estimated read count per single mRNA molecule (of type i) is

$$\widetilde{TRC_i} = \frac{TRC_i}{M_i}$$

The total sum of reads for the entire transcriptome is:

$$TRC_{system} = \sum_{i=1}^{N} TRC_i$$

Suppose the total number of ribosomes at the pool is G_{tot}. At steady state, roughly $\alpha = 80\%$ of the ribosomes are active (8, 16), i.e. are involved in a translation elongation process. We define a normalization factor that is data-specific (i.e. can be different for different ribosomal sequencing experiments):

$$\eta = \frac{\alpha G_{tot}}{TRC_{system}}$$

which means ribosomes per read. We then can multiply $\widetilde{TRC_i}$ by η to get the expected absolute number of ribosomes on each mRNA of type i:

$$g_i = \eta \widetilde{TRC_i}$$

For each mRNA i we perform a single-molecule DTASEP simulation in order to find the initiation time that results in g_i ribosomes along the ORF in steady state. For the decoding times we use the Typical Decoding Rates as estimated and published by Dana et al. However, these values are correlated to the actual decoding times and need to be calibrated. Various studies indicate the average peptide chain elongation rate: 8-15 aa/sec (17), 12-17 aa/sec (18), 1.7-5.0 aa/sec (19), 4.2-21.6 (15), 12-22 aa/sec (8), 8.3 aa/sec (20) and 22 aa/s (21). The values are growth-rate dependent (values reported are usually in the range of 0.4-2 doublings/hour) and temperature (usually 25-37C). In some cases, the estimation were done on an *in-vitro* system or on small amount of genes. In general, recent studies seem to agree on higher values for prokaryotic cells. For our analysis, we use the mid-high value of 18 aa/sec (resulting in calibration factor of 3.41 to the original Typical Decoding Times). Also note that calculating the average decoding time requires taking into account both the ORF composition of every gene and the corresponding mRNA abundance.

For a given average elongation rate and a ribosomal pool value G_{tot}, a range of initiation rates will be obtained using the method described above. These values must also agree with reported values.

10. Estimation of termination time

The termination time was estimated based on ribosome profiling of the stop codon region of *E. coli* mRNAs (22), relying on the assumption the higher count at a given position indicates a higher dwell time. Using figure 2.A (in (22)) we estimated that the ribosomal dwell time at the stop codon is 2.5 times higher than the average dwell time at nucleotides 21-45 to the left of the stop codon (i.e. at codons 8-15, counting from the stop codon as zero to 5' direction). Using all *E. coli* genes and the previously estimated decoding times, the genomic average of dwell times at the discussed codons is 0.13, leading to estimation termination time of 0.33s.

11. Parameter robustness tests

After choosing the nominal parameters for the system, each parameter was tested for robustness by performing a set of simulations with values around the chosen value. In the case of tRNA pool, since the selected value was in the saturation region of observed metrics, we focused on lower values. The test was qualitative, while making sure that the trends are as expected. We also made sure that no rapid changes were observed in the region of the selected values.

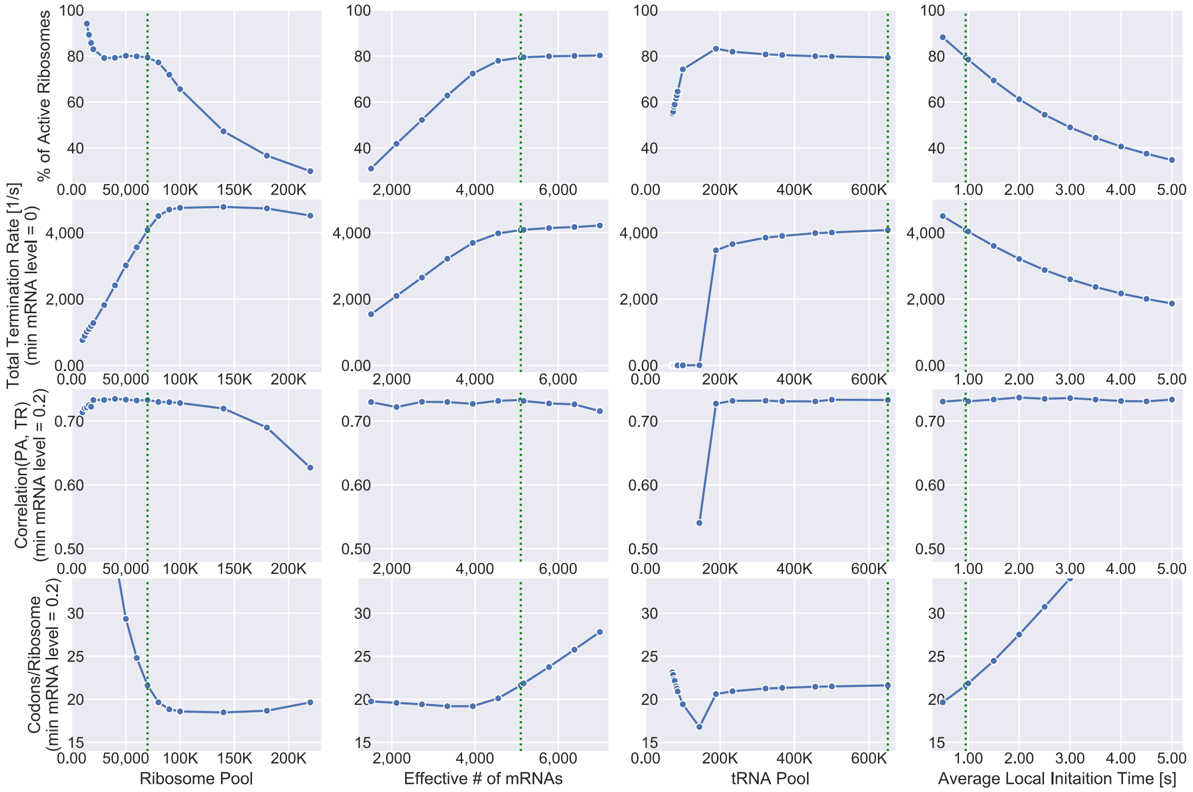

Fig J: Robustness tests of the primary model parameters: ribosome pool, effective number of mRNAs, tRNA pool and the average local initiation time. All parameters found to be robust from the standpoint of the chosen metrics and showed expected behaviour.

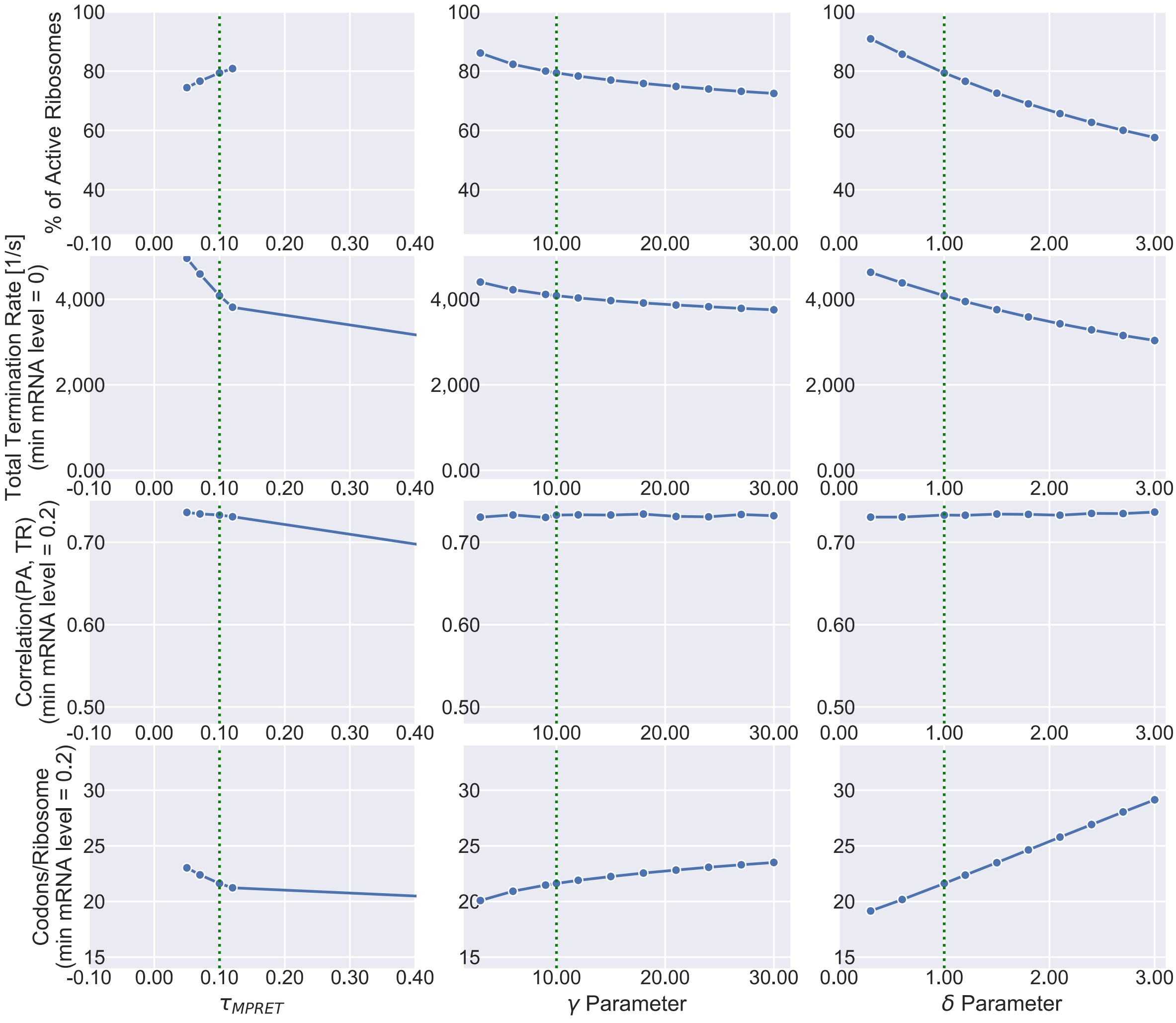

Fig K: Robustness tests of the secondary model parameters: τ_{MPRET}, γ and δ. All parameters found to be robust from the standpoint of the chosen metrics.

12. Details of GFP heterologous expression modelling

We used the following GFP sequence (from the U57608.1 cloning vector):

```
ATGGTGAGCAAGGGCGAGGAGCTGTTCACCGGGGTGGTGCCCATCCTGGTCGAGCTGGACGGCGACGTAAACGGCCACAAGTTCAGCGTGTCCGGCGAGG
GCGAGGGCGATGCCACCTACGGCAAGCTGACCCTGAAGTTCATCTGCACCACCGGCAAGCTGCCCGTGCCCTGGCCCACCCTCGTGACCACCCTGACCTA
CGGCGTGCAGTGCTTCAGCCGCTACCCCGACCACATGAAGCAGCACGACTTCTTCAAGTCCGCCATGCCCGAAGGCTACGTCCAGGAGCGCACCATCTTC
TTCAAGGACGACGGCAACTACAAGACCCGCGCCGAGGTGAAGTTCGAGGGCGACACCCTGGTGAACCGCATCGAGCTGAAGGGCATCGACTTCAAGGAGG
ACGGCAACATCCTGGGGCACAAGCTGGAGTACAACTACAACAGCCACAACGTCTATATCATGGCCGACAAGCAGAAGAACGGCATCAAGGTGAACTTCAA
GATCCGCCACAACATCGAGGACGGCAGCGTGCAGCTCGCCGACCACTACCAGCAGAACACCCCCATCGGCGACGGCCCCGTGCTGCTGCCCGACAACCAC
TACCTGAGCACCCAGTCCGCCCTGAGCAAAGACCCCAACGAGAAGCGCGATCACATGGTCCTGCTGGAGTTCGTGACCGCCGCCGGGATCACTCTCGGCA
TGGACGAGCTGTACAAGTAA
```

The assumption that this cloning vector is highly optimized for expression in *E. coli*, led to the following additional two assumptions:

1. The amount of GFP mRNA molecules was assumed be very high, e.g. 20% of all mRNA molecules. We thus introduced 1,275 GFP mRNAs (in additional to the 5,100 native RNAs). Note that increasing this fraction further resulted in resources depletion.
2. The initiation rate had to be high in comparison to initiation rates of the native genes. We calculated the mean initiation rate of the 10 genes with the highest initiation rates and defined the result as the initiation rate of all GFP genes.

13. Initiation time estimation of Kudla et. al variants

In a heterologous expression simulation, it is required to define the number of mRNAs, the initiation time and the codon composition of each mRNA. In this case, all the heterologous mRNAs in a single simulation are from the same type, which is one of the variants analyzed by Kudla *et al*.

The baseline initiation time τ_h for all variants was defined as the mean value of 10 lowest initiation time values of the native mRNAs. As in section 12, this definition reflects the assumption that the used vector and promoter are generally optimized for the selected host and thus have a relatively high initiation rate. The actual initiation rate was defined as $\tau_h \exp\left(-\frac{FE}{c_h}\right)$, where FE is the folding energy on the first third of the ORF, to reflect the idea that initiation time is locally affected by the strength of the secondary structure folding. In order to calibrate c_h, we have chosen 10 variants (sampled uniformly by first sorting all variants by FE and then by optical density), and performed simulations for these variants for various values of c_h. The chosen value, $c_h = 8$, was chosen for maximizing the correlation between PA and the predicted termination rate of GFP mRNAs. Assuming that the reported mRNA level of the i-th variant is m_i, the mRNA level we defined for the simulation was μm_i, where $\mu = \frac{1275}{\langle m_i \rangle}$. This approach, that was used for both calibration and main simulations, forces the average number of mRNAs to be 20% of the total amount (as suggested in section 12), but maintains the relative variations of mRNA levels between variants.

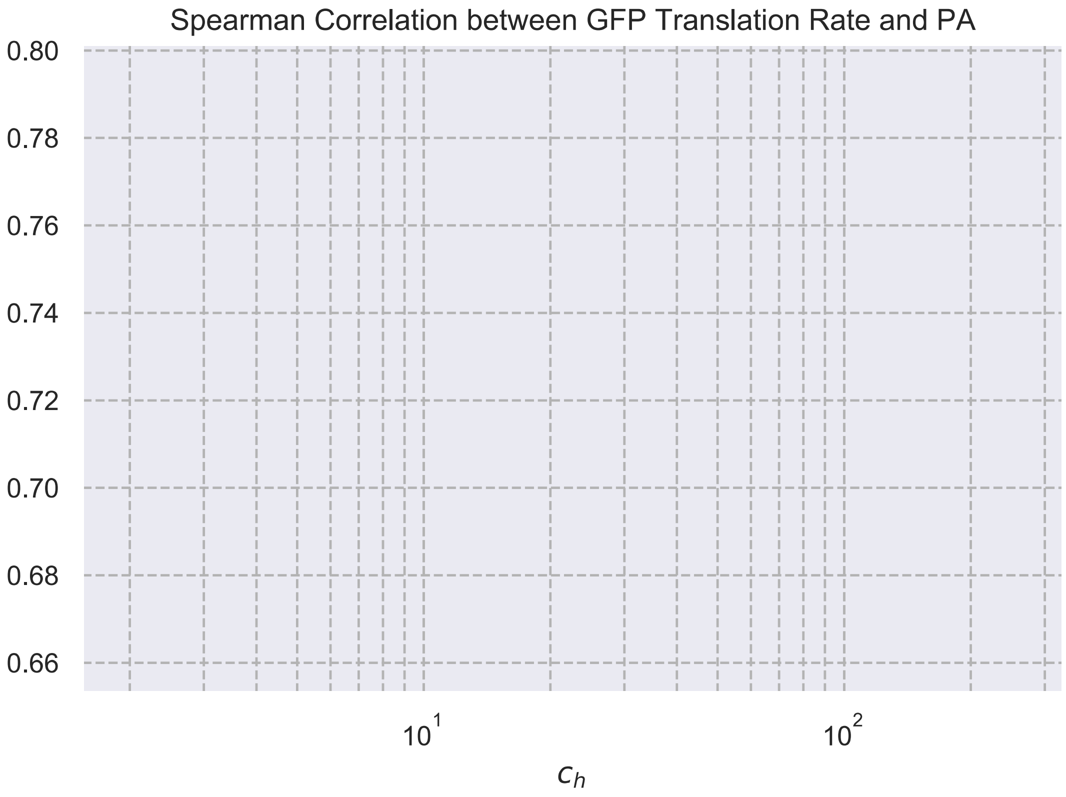

Fig L: Optimization of the c_h coefficient. We performed simulation for 10 variants (that uniformly represent PA and OD of the entire set) and calculated correlation between PA and the predicted GFP translation rate for various values of c_h. The value $c_h = 8$ was chosen.

14. Correlation between read count and local initiation rate

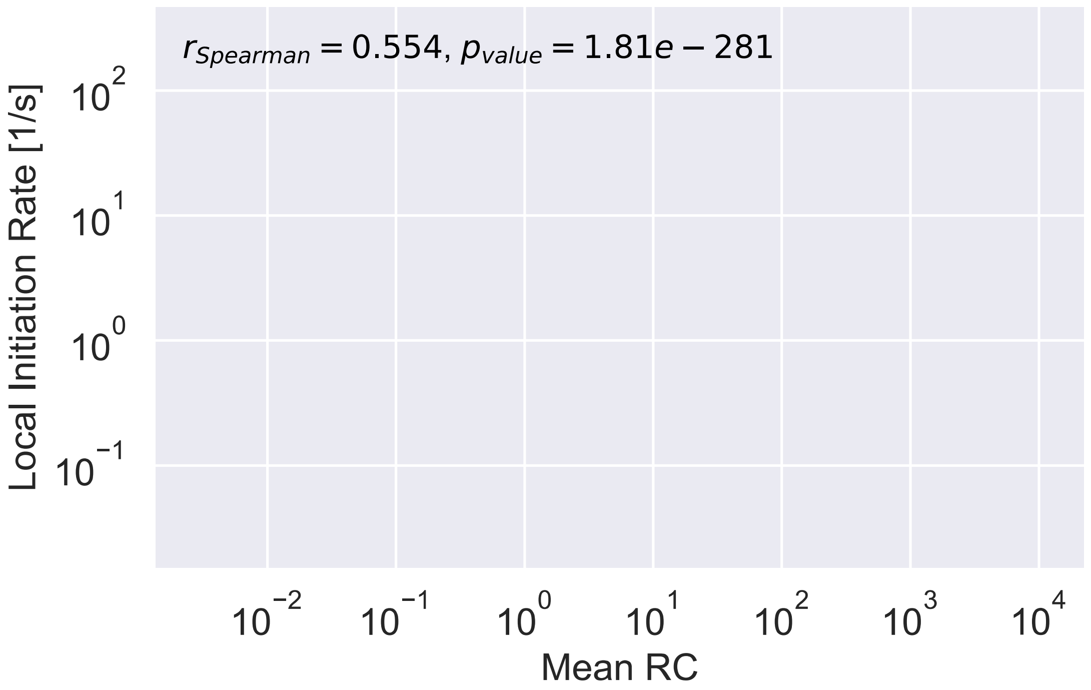

Fig M: Local initiation rate vs. mean RC (read count) for all available *E. coli* genes.

15. Correlations between model predictions and Kudla et. al data

Below we show correlations between PA (protein abundance) and OD (optical density) (23), and non-experimental values, such as FE (folding energy of the first third of the ORF) and prediction of the model.

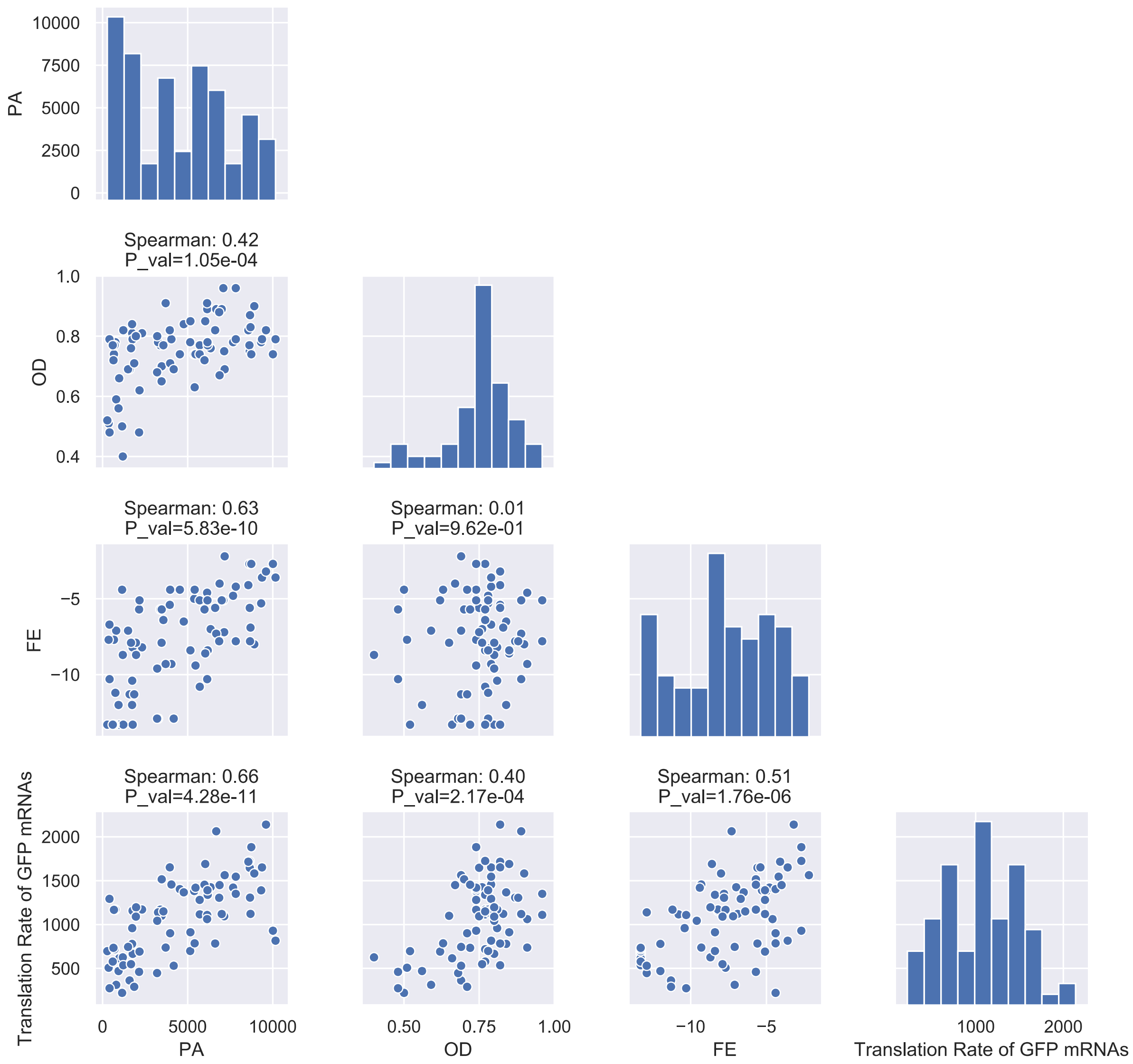

Fig N: Correlation between pairs from the group {PA, OD, FE, TR}, where TR stands for the translation rate of GFP proteins.

16. Regressors for PA and OD, simple features

Based on 77 variants for which all data is available.

PA prediction:

Variables	r_Spearman	p_value	r_Pearson	p_value	Estimated parameters (1st is intercept)
FE	0.62	2.36E-09	0.62	1.61E-09	8898.738,579.651
active_ribosomes	0.7	1.56E-12	0.7	1.58E-12	-71514.109,90321.861
GFP_tr	0.64	2.44E-10	0.65	1.76E-10	-408.636,4.585
CAI	0.13	2.78E-01	0.08	4.70E-01	3601.393,2582.032
Num_of_GFPs	0.52	1.61E-06	0.51	2.33E-06	894.84,2.855
FE,active_ribosomes	0.74	2.67E-14	0.72	1.09E-13	-49847.136,243.124,66782.611
FE,GFP_tr	0.73	3.14E-14	0.73	5.71E-14	3902.94,362.889,3.151
FE,CAI	0.67	2.90E-11	0.65	1.38E-10	7186.863,612.092,6135.387

FE,Num_of_GFPs	0.73	4.19E-14	0.72	2.07E-13	5620.066,489.173,2.086
active_ribosomes,GFP_tr	0.71	6.29E-13	0.7	1.14E-12	-93483.402,118470.173,-1.609
active_ribosomes,CAI	0.72	1.03E-13	0.72	2.29E-13	-74963.073,92540.551,4951.503
active_ribosomes,Num_of_GFPs	0.72	9.14E-14	0.71	3.22E-13	-94085.747,119377.921,-1.502
GFP_tr,CAI	0.65	1.85E-10	0.65	1.35E-10	-1016.482,4.571,1947.324
GFP_tr,Num_of_GFPs	0.72	2.76E-13	0.72	2.58E-13	-470.526,10.532,-5.021
CAI,Num_of_GFPs	0.52	1.40E-06	0.52	1.62E-06	70.054,2579.329,2.855
FE,active_ribosomes,GFP_tr	0.73	3.14E-14	0.73	5.70E-14	2052.963,358.36,2286.25,3.05
FE,active_ribosomes,CAI	0.76	7.39E-16	0.75	5.98E-15	-50843.949,277.949,66048.589,5886.753
FE,active_ribosomes,Num_of_GFPs	0.73	5.59E-14	0.72	1.05E-13	-39879.537,286.208,54722.758,0.408
FE,GFP_tr,CAI	0.76	1.47E-15	0.74	1.10E-14	2927.108,398.507,2.978,4481.943
FE,GFP_tr,Num_of_GFPs	0.74	2.39E-14	0.73	4.16E-14	2815.698,273.159,5.537,-1.715
FE,CAI,Num_of_GFPs	0.76	6.72E-16	0.74	1.58E-14	4135.198,520.985,5604.555,2.036
active_ribosomes,GFP_tr,CAI	0.74	1.28E-14	0.73	3.11E-14	-135497.985,168966.173,-4.304,7506.026
active_ribosomes,GFP_tr,Num_of_GFPs	0.72	1.82E-13	0.72	1.05E-13	-47122.119,59401.658,5.826,-3.67
active_ribosomes,CAI,Num_of_GFPs	0.74	1.16E-14	0.74	1.99E-14	-103091.576,128337.938,5869.815,-1.829
GFP_tr,CAI,Num_of_GFPs	0.72	1.04E-13	0.72	2.32E-13	-823.884,10.461,1134.153,-4.967

OD prediction:

Variables	r_Spearman	p_value	r_Pearson	p_value	Estimated parameters (1st is intercept)
FE	-0.01	9.12E-01	0.06	5.79E-01	0.767,0.002
active_ribosomes	0.31	6.16E-03	0.37	8.98E-04	-0.765,1.802
GFP_tr	0.38	7.29E-04	0.5	4.43E-06	0.611,0.0
CAI	0.6	8.15E-09	0.53	6.96E-07	0.553,0.616
Num_of_GFPs	0.39	5.29E-04	0.49	7.07E-06	0.623,0.0
FE,active_ribosomes	0.33	3.14E-03	0.46	2.55E-05	-1.954,-0.013,3.094
FE,GFP_tr	0.41	1.88E-04	0.55	2.79E-07	0.5,-0.009,0.0
FE,CAI	0.53	7.37E-07	0.55	1.73E-07	0.586,0.006,0.649
FE,Num_of_GFPs	0.4	3.68E-04	0.49	5.68E-06	0.6,-0.002,0.0
active_ribosomes,GFP_tr	0.5	4.56E-06	0.61	3.88E-09	5.242,-5.895,0.0
active_ribosomes,CAI	0.6	9.51E-09	0.68	7.77E-12	-1.232,2.103,0.67
active_ribosomes,Num_of_GFPs	0.4	3.28E-04	0.49	5.47E-06	1.127,-0.634,0.0
GFP_tr,CAI	0.63	9.82E-10	0.72	2.72E-13	0.424,0.0,0.599
GFP_tr,Num_of_GFPs	0.37	7.91E-04	0.5	3.70E-06	0.612,0.0,0.0
CAI,Num_of_GFPs	0.64	3.76E-10	0.72	1.57E-13	0.426,0.616,0.0
FE,active_ribosomes,GFP_tr	0.51	2.60E-06	0.62	2.25E-09	6.967,0.006,-7.993,0.001
FE,active_ribosomes,CAI	0.62	1.68E-09	0.71	4.92E-13	-2.062,-0.01,3.014,0.638
FE,active_ribosomes,Num_of_GFPs	0.4	3.30E-04	0.49	5.47E-06	1.106,-0.0,-0.609,0.0
FE,GFP_tr,CAI	0.64	5.33E-10	0.72	9.59E-14	0.376,-0.005,0.0,0.568
FE,GFP_tr,Num_of_GFPs	0.44	5.87E-05	0.58	2.76E-08	0.367,-0.02,0.0,-0.0
FE,CAI,Num_of_GFPs	0.63	6.68E-10	0.72	1.45E-13	0.435,0.001,0.623,0.0
active_ribosomes,GFP_tr,CAI	0.64	3.28E-10	0.73	7.55E-14	2.334,-2.4,0.0,0.52
active_ribosomes,GFP_tr,Num_of_GFPs	0.53	7.79E-07	0.64	3.51E-10	7.181,-8.365,0.001,-0.0
active_ribosomes,CAI,Num_of_GFPs	0.63	5.57E-10	0.72	1.43E-13	0.169,0.319,0.624,0.0
GFP_tr,CAI,Num_of_GFPs	0.64	4.68E-10	0.72	1.15E-13	0.422,0.0,0.609,0.0

17. Correlations between PA (and OD) and ESDR per codon

The data is given in a separate excel spreadsheet.

S2_File.xlsx

18. Regressors for PA and OD, based of ESDR features

We list the 4 combinations discussed in the main text. In each case, we list the 20 chosen features. It is not surprising that FE or CAI are chosen as the first feature, since these metric are tightly related to the translational efficiency of given gene. The other features are codon specific, thus they are expected to contribute when sufficient amount of them is combined together.

Objective: PA, features set: ESDR per codon, CAI and FE

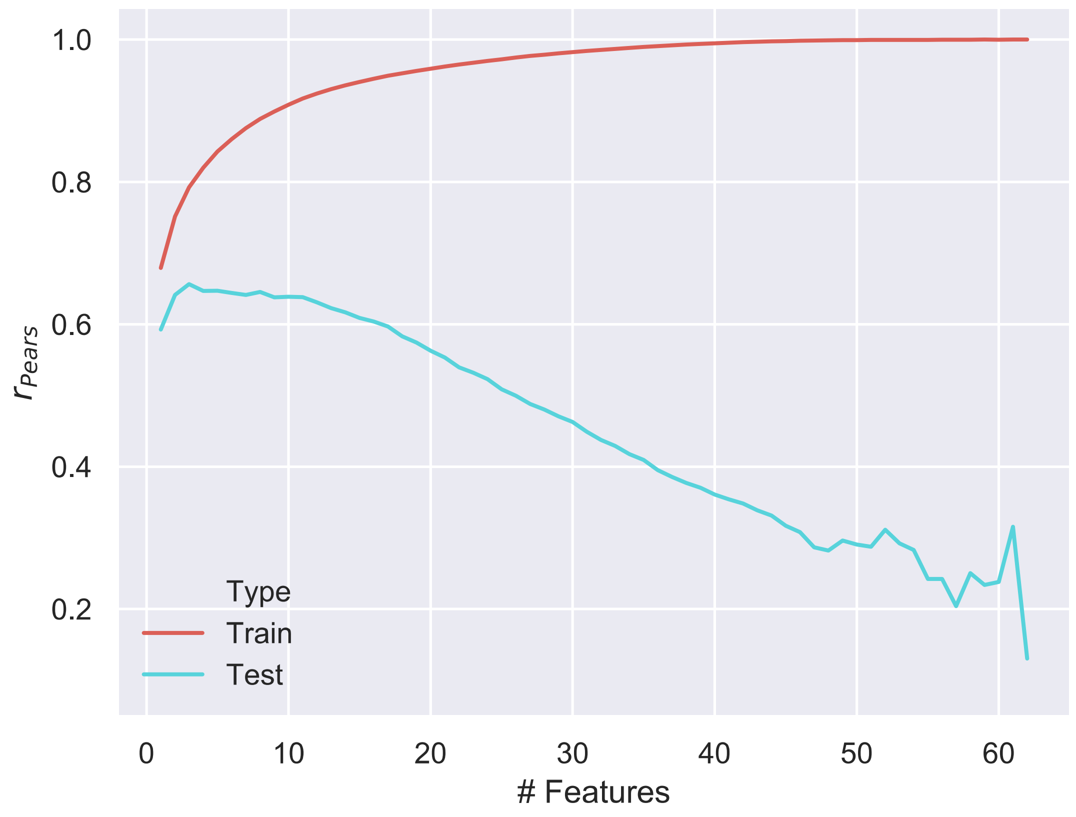

Fig O: Summary of the feature selection procedure when predicting PA using a feature set based on ESDR per codon.

List of 20 best features, ordered: FE, ESDR_CAC, ESDR_UGG, ESDR_CGA, ESDR_UAU, ESDR_CUU, ESDR_GUA, ESDR_UCU, ESDR_ACU, ESDR_CGU, ESDR_CUA, ESDR_AAA, ESDR_CUC, ESDR_AGU, ESDR_GAU, ESDR_GGU, ESDR_GCA, ESDR_GCG, ESDR_AUA, ESDR_GUG

Objective: OD, features set: ESDR per codon, CAI and FE

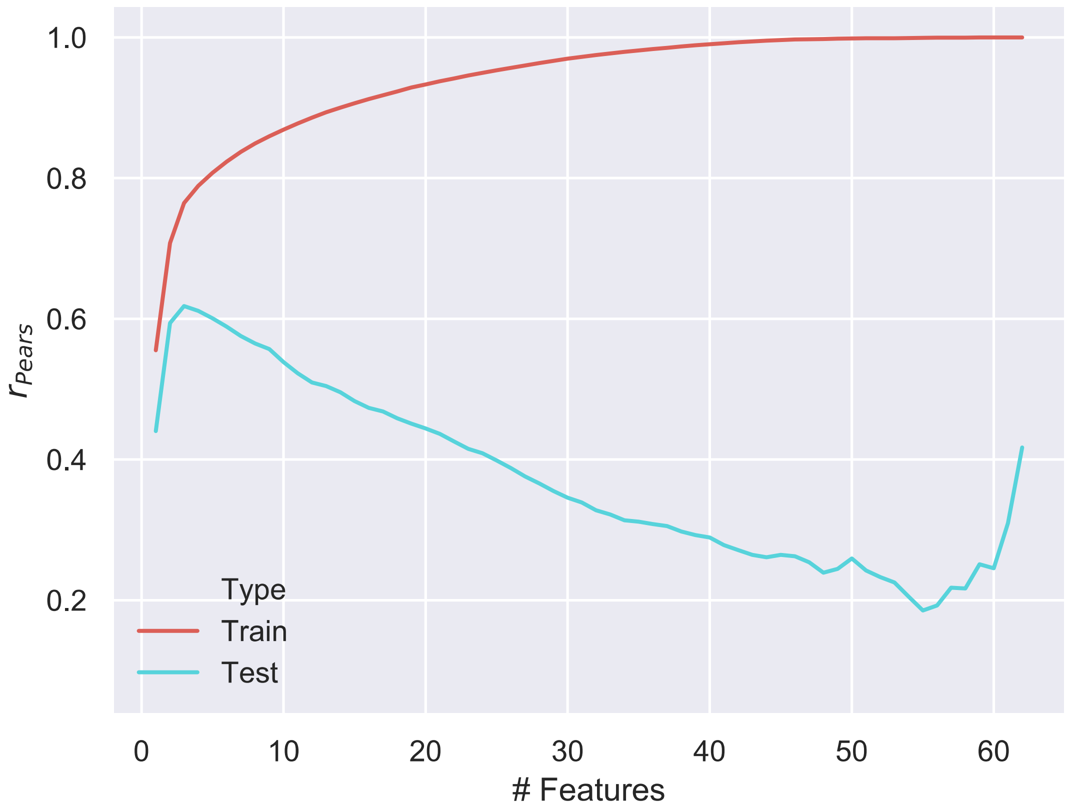

Fig P: Summary of the feature selection procedure when predicting OD using a feature set based on ESDR per codon.

List of 20 best features, ordered: CAI, ESDR_UAU, ESDR_AGA, ESDR_CAA, ESDR_CGU, ESDR_GUC, ESDR_CGA, ESDR_AGU, ESDR_GCA, ESDR_CGG, ESDR_GGG, ESDR_CUA, ESDR_GAU, ESDR_UGU, ESDR_CUG, ESDR_UGG, ESDR_CAC, ESDR_AUU, ESDR_ACA, ESDR_UCC

Objective: PA, features set: count per codon, CAI and FE

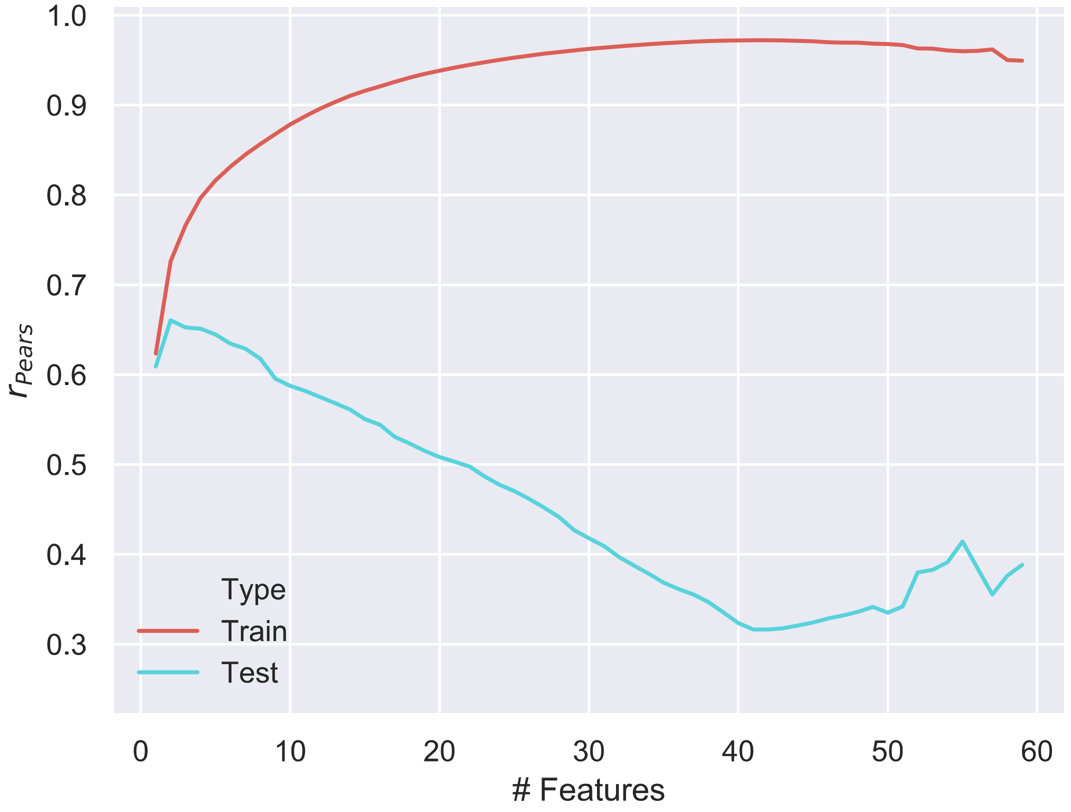

Fig Q: Summary of the feature selection procedure when predicting PA using a feature set based on count per codon.

List of 20 best features, ordered: FE, count_ACA, count_AUU, count_ACU, count_UCA, count_GUC, count_CGU, count_CUU, count_GGG, count_CUA, count_AAA, count_CAG, count_GGC, count_CGG, count_GUG, count_UCU, count_AAC, count_UGC, count_ACG, count_AGA

<u>Objective: OD, features set: count per codon, CAI and FE</u>

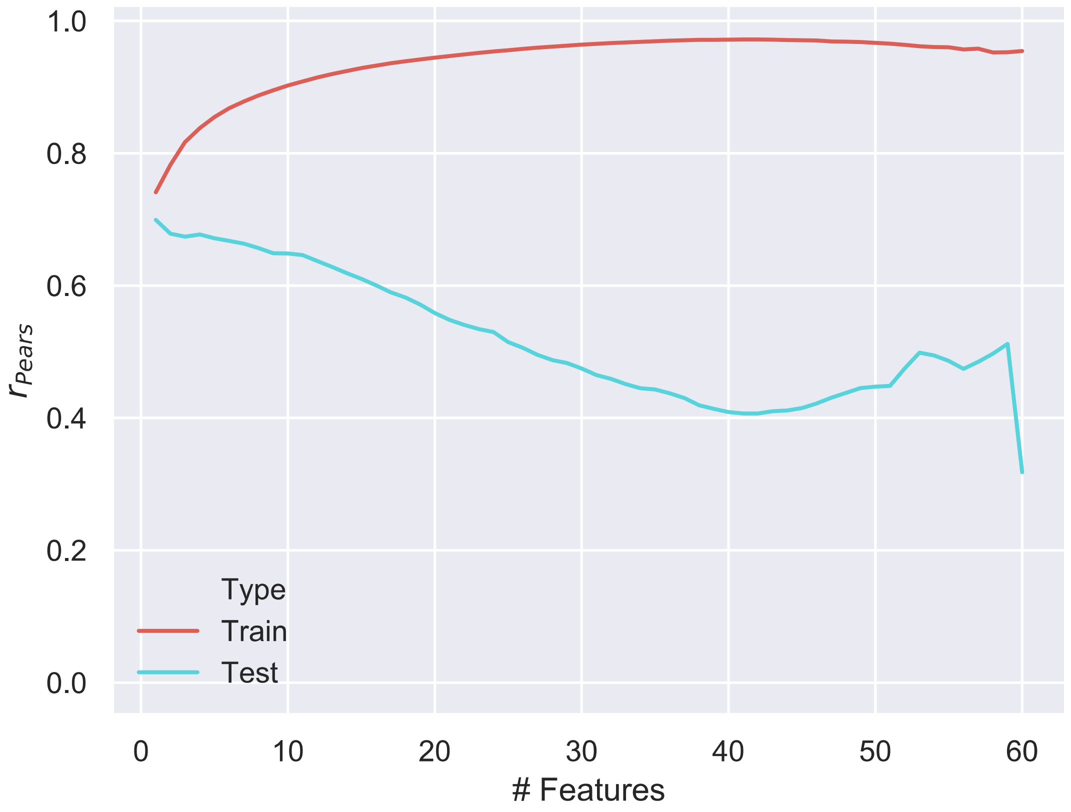

Fig R: Summary of the feature selection procedure when predicting OD using a feature set based on count per codon.

List of 20 best features, ordered: count_UCU, count_CGU, count_CUA, count_ACU, count_CGA, count_AAU, count_UUU, count_CAC, count_CGG, count_GCG, count_AAC, count_UCG, count_CUU, count_GGG, count_CAU, count_UCC, count_GGU, count_CGC, count_ACA, count_UCA

19. References

1. Dong,H., Nilsson,L. and Kurland,C.G. (1996) Co-variation of tRNA abundance and codon usage in Escherichia coli at different growth rates. *J. Mol. Biol.*, 10.1006/jmbi.1996.0428.

2. Blattner,F.R., Plunkett,G., Bloch,C.A., Perna,N.T., Burland,V., Riley,M., Collado-Vides,J., Glasner,J.D., Rode,C.K., Mayhew,G.F., *et al.* (1997) The complete genome sequence of Escherichia coli K-12. *Science (80-.).*, 10.1126/science.277.5331.1453.

3. Sharp,P.M. and Li,W.H. (1987) The codon adaptation index-a measure of directional synonymous codon usage bias, and its potential applications. *Nucleic Acids Res.*, 10.1093/nar/15.3.1281.

4. dos Reis,M., Savva,R. and Wernisch,L. (2004) Solving the riddle of codon usage preferences: A test for translational selection. *Nucleic Acids Res.*, 10.1093/nar/gkh834.

5. Dana,A. and Tuller,T. (2014) The effect of tRNA levels on decoding times of mRNA codons. *Nucleic Acids Res.*, **42**, 9171–9181.

6. Plotkin,J.B. and Kudla,G. (2011) Synonymous but not the same: The causes and consequences of codon bias. *Nat. Rev. Genet.*, 10.1038/nrg2899.

7. Borg,A. and Ehrenberg,M. (2015) Determinants of the rate of mRNA translocation in bacterial protein synthesis. *J. Mol. Biol.*, 10.1016/j.jmb.2014.10.027.

8. Bremer,H. and Dennis,P.P. (1996) Modulation of Chemical Composition and Other Parameters of the Cell by Growth Rate. *Escherichia coli Salmonella Cell. Mol. Biol.*, **2**, 1527–1542.

9. Li,G.W., Oh,E. and Weissman,J.S. (2012) The anti-Shine-Dalgarno sequence drives translational pausing and codon choice in bacteria. *Nature*, 10.1038/nature10965.

10. Bartholomäus,A., Fedyunin,I., Feist,P., Sin,C., Zhang,G., Valleriani,A. and Ignatova,Z. (2016) Bacteria differently regulate mRNA abundance to specifically respond to various stresses. *Philos. Trans. R. Soc. A Math. Phys. Eng. Sci.*, 10.1098/rsta.2015.0069.

11. Sezonov,G., Joseleau-Petit,D. and D'Ari,R. (2007) Escherichia coli physiology in Luria-Bertani broth. *J. Bacteriol.*, 10.1128/JB.01368-07.

12. Milo,R., Jorgensen,P., Moran,U., Weber,G. and Springer,M. (2009) BioNumbers The database of key numbers in molecular and cell biology. *Nucleic Acids Res.*, 10.1093/nar/gkp889.

13. Großmann,P., Lück,A. and Kaleta,C. (2017) Model-based genome-wide determination of RNA chain elongation rates in Escherichia coli. *Sci. Rep.*, 10.1038/s41598-017-17408-9.

14. Laursen,B.S., Sørensen,H.P., Mortensen,K.K. and Sperling-Petersen,H.U. (2005) Initiation of protein synthesis in bacteria. *Microbiol. Mol. Biol. Rev.*, 10.1128/MMBR.69.1.101-123.2005.

15. Sørensen,M.A. and Pedersen,S. (1991) Absolute in vivo translation rates of individual codons in Escherichia coli. The two glutamic acid codons GAA and GAG are translated with a threefold difference in rate. *J. Mol. Biol.*, 10.1016/0022-2836(91)90211-N.

16. Forchhammer,J. and Lindahl,L. (1971) Growth rate of polypeptide chains as a function of the cell growth rate in a mutant of Escherichia coli 15. *J. Mol. Biol.*, 10.1016/0022-2836(71)90337-8.

17. Talkad,V., Schneider,E. and Kennell,D. (1976) Evidence for variable rates of ribosome movement in Escherichia coli. *J. Mol. Biol.*, 10.1016/0022-2836(76)90015-2.

18. Young,R. and Bremer,H. (1976) Polypeptide-chain-elongation rate in Escherichia coli B/r as a function of growth rate. *Biochem. J.*, 10.1042/bj1600185.

19. Pedersen,S. (1984) Escherichia coli ribosomes translate in vivo with variable rate. *EMBO J.*, 10.1002/J.1460-2075.1984.TB02227.X.

20. Golding,I., Paulsson,J., Zawilski,S.M. and Cox,E.C. (2005) Real-time kinetics of gene activity in individual bacteria. *Cell*, 10.1016/j.cell.2005.09.031.

21. Johansson,M., Bouakaz,E., Lovmar,M. and Ehrenberg,M. (2008) The Kinetics of Ribosomal Peptidyl Transfer Revisited. *Mol. Cell*, 10.1016/j.molcel.2008.04.010.

22. Baggett,N.E., Zhang,Y. and Gross,C.A. (2017) Global analysis of translation termination in E. coli. *PLoS Genet.*, 10.1371/journal.pgen.1006676.

23. Kudla,G., Murray,A.A.W., Tollervey,D. and Plotkin,J.B.J.B. (2009) Coding-sequence determinants of gene expression in Escherichia coli. *Science (80-.).*, **324**, 255–258.
